# Boom‐Bust Dynamics Drive Community‐Wide Dietary Structuring in Desert‐Dwelling Raptors

**DOI:** 10.1002/ece3.73002

**Published:** 2026-01-30

**Authors:** Rhys J. Cairncross, Christopher R. Dickman

**Affiliations:** ^1^ School of Life and Environmental Sciences The University of Sydney Camperdown New South Wales Australia

**Keywords:** arid bioregion, Australia, diet, productivity boom, pulse‐reserve, rodent irruption

## Abstract

Boom‐bust dynamics (pulses of productivity followed by extended periods of low productivity) characterise many dryland, arid environments. These dynamics drive diverse ecological functions, with populations and communities of biota responding strongly to temporal changes in productivity. These changes have flow‐on effects to dietary structuring for consumers, including top predators. Here, we aimed to assess the influence of rainfall‐triggered productivity changes on the diets of raptors using data from long‐term monitoring of a diverse (19‐species) raptor community in a central Australian desert subject to extreme boom‐bust conditions. We showed that boom‐bust dynamics influence dietary structure of the raptor community and that dietary responses are mediated by species‐specific prey preferences and movement ecology. Mean raptor dietary niche breadth increased in bust (0.012 ± 0.0007 [SE]) compared to boom (0.009 ± 0.0006) periods (model *β* = 0.350) whilst overlap between raptor diets increased in boom versus bust periods. Small mammals, mostly irruptive rodents, and birds featured more in the diets of raptors during boom periods whereas reptiles were prominent during busts. Sedentary, resident, raptors had more varied but consistent diets between boom and bust periods in comparison to locally nomadic and nomadic species. Our results provide the first elucidation of dietary structure and its interaction with boom‐bust dynamics in a highly diverse assemblage of desert raptors. Except for dietary specialists, we propose that predators generally focus on preferred prey when productivity peaks but expand their diets to include alternative prey when conditions are unfavourable. Dietary switching may aid the persistence of such raptor species as boom‐bust systems destabilise due to climate change, whereas dietary specialists, especially if sedentary, are likely to be at risk if preferred prey taxa decline for long periods under future climates.

## Graphical abstract

We investigated how boom‐bust productivity dynamics affected dietary structuring within raptor communities of an arid bioregion. We found that higher productivity was associated with high dietary overlap, lower niche breadth and greater foraging on small mammals; however, raptors that were nomadic exhibited greater specialisation regardless of productivity. We suggest that as climate change alters rainfall‐driven productivity cycles and associated prey communities, raptors with broader diets will be more resilient whereas those with narrower diets, especially those that are nomadic, will be more threatened.

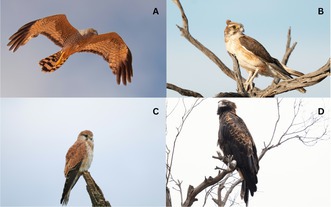

## 1 Introduction

Arid environments are unique systems with ecological dynamics commonly underpinned by the pulse‐reserve paradigm of primary productivity (Yang et al. 2010). Many animal populations in these environments exhibit dramatic ‘booms’ in numbers during periods of peak productivity before declining during ‘bust’ periods when productivity declines (Chesson et al. 2004; Yang et al. 2010). Rainfall is the major driver of these dynamics since water is usually limited in arid environments (Holmgren et al. 2006), with large rainfall events resulting in elevated plant growth and reproduction (Dickman et al. 2014; Greenville et al. 2014; Heisler‐White et al. 2008; Letnic and Dickman 2010; Wardle et al. 2013). Increased productivity drives greater abundance and richness of consumers such as insects (Kwok et al. 2016), birds (Jordan et al. 2017; Pavey and Nano 2013; Seymour et al. 2015; Smith 2015; Tischler et al. 2013), mammals (Bennison et al. 2018; Letnic et al. 2005; Letnic and Dickman 2006) and reptiles (Dickman, Letnic, and Mahon 1999; Greenville et al. 2016b; Read et al. 2012; Schlesinger et al. 2010), although species responses differ, varying in timing and magnitude (Greenville et al. 2016a, 2016b; Pastro et al. 2013; Pavey and Nano 2009). Boom‐bust ecosystems are well known in Australia's central deserts, which are home to diverse faunal communities strongly affected by rainfall pulses (Morton 2022). For example, irruptions in rodent populations commonly follow large rainfall events (Dickman, Mahon, et al. 1999), triggering in situ increases in predator populations such as feral cats ( *Felis catus* ) and red foxes ( *Vulpes vulpes* ) (Greenville et al. 2014), and influxes of mobile predators such as raptors (Pavey 2021).

Uncovering predator–prey relationships and their contribution to food web structuring is a key challenge in ecology (Nielsen et al. 2018), with the role of predation particularly prominent in arid ecosystems (Ayal 2007). Dietary studies have been undertaken for a range of predators in arid regions (Pavey et al. 2008; Vernes et al. 2021; Yip et al. 2015; Yip and Dickman 2023), including raptors (Aumann 2001), but the influence of boom‐bust prey dynamics on the dietary structuring of raptor communities remains uncertain. Such knowledge is important for informing how foraging and predation change with environmental conditions, since this may affect the persistence and survival of threatened species (Spencer et al. 2017; Tatler et al. 2019; Yip et al. 2015) as well as community trophic relationships and niche partitioning (Pavey et al. 2008; Porter et al. 2022). Raptors are declining in many regions (McClure et al. 2018; O'Bryan et al. 2025), including arid regions of Australia (Gardner et al. 2022), so elucidating these relationships among desert raptors may be instructive for conservation by bridging knowledge gaps in their ecology (Buechley et al. 2019).

The diets of raptors are influenced by environmental factors (Charter et al. 2018; Porter et al. 2022), with species exploiting prey in different ways depending on their hunting strategies, diel activity, prey preferences and habitat usage (Dickman et al. 1991; Jaksic 1985; Piana and Marsden 2012; Robinson 1994; Steenhof and Kochert 1988). Thus, raptor communities are often structured by differences in diet. Avian communities typically show more dietary overlap during periods of high resource availability, with greater partitioning occurring when resources are limited (Porter et al. 2022). Indeed, there is evidence that raptors overlap most in diet when preferred food sources are plentiful (Charter et al. 2018). For instance, in arid regions of Australia, irruptions of rodents have been linked to high dietary overlap between letter‐winged kites ( *Elanus scriptus* ) and barn owls ( *Tyto alba* ) (Pavey et al. 2008), despite these birds sharing similar nocturnal foraging strategies. Conversely, dietary niche breadth may increase during periods when primary prey items diminish, as a wider diversity of alternative prey is eaten (Steenhof and Kochert 1988).

Temporal fluctuations in prey, and changes in the diets of raptors and other predators, are difficult to observe in the absence of long‐term monitoring (Ponce et al. 2018), especially in desert landscapes driven by extreme boom‐bust cycles. Many arid regions are also visited by migrant raptors only during boom periods, resulting in transient but dramatic increases in predator density (Vidal‐Mateo et al. 2022). Although the diets of these raptors may be partitioned (Buij et al. 2013; Gangoso et al. 2013), they are likely to be affected by boom periods of higher productivity. For instance, ecologically specialised raptors may be nomadic if favoured prey are not consistently present at a local level, and move constantly in search of food (Gangoso et al. 2013). However, if environmental conditions are favourable, their movements and foraging strategies change (Foysal and Panter 2024a, 2024b; Pavey 2021). By contrast, the diets of sedentary species are likely to be more invariant and include a range of prey types that are available at all stages of the boom and bust cycle. Overall, while we have some understanding of how these dynamics interact with the diets of arid‐dwelling mammalian carnivores (Tatler et al. 2019), we have less information for raptors, with implications for understanding the persistence of these birds.

**FIGURE 1:**
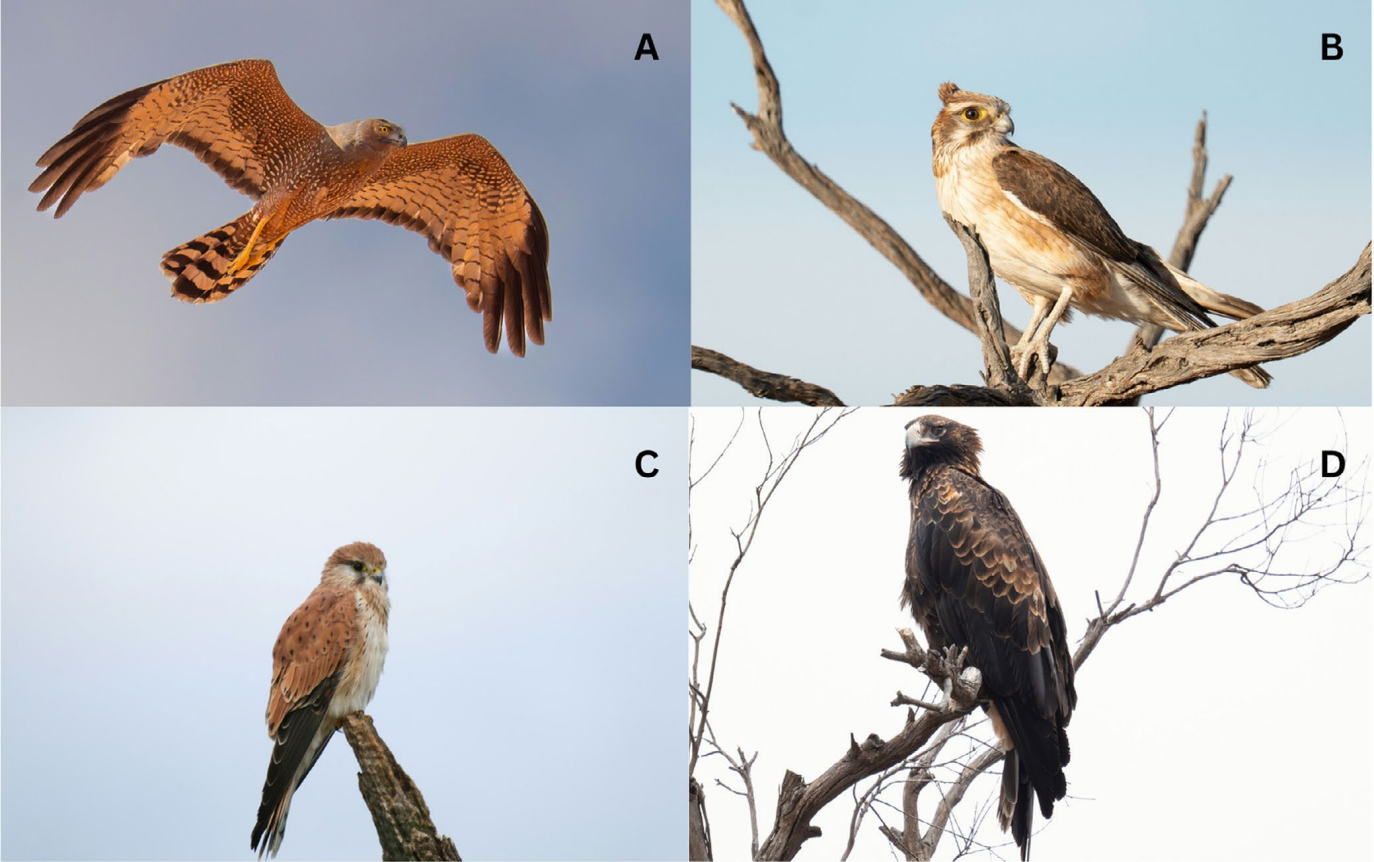
Examples of species within the raptor community of study. (A) Spotted harrier ( *Circus approximans* ). (B) Brown falcon ( *Falco berigora* ). (C) Nankeen kestrel ( *F. cenchroides* ). (D) Wedge‐tailed eagle ( *Aquila audax* ). Photo credits: A, B, D—lead author; C—Niraj Meisuria.

In this study, we ask how boom‐bust dynamics drive community‐wide dietary structuring in a diverse assemblage of Australian desert raptors (Figure 1), addressing this question by testing five hypotheses. We predicted that niche overlap between species would increase in boom periods (Pavey et al. 2008), since there will be greater numbers of preferred prey items and more raptor species to hunt them, while niche breadth would increase in bust periods as raptors exploit a wider diversity of prey (H1). Next, we predicted that the prey items in the diets of raptors would differ between species and species groups across boom and bust periods (H2), since dietary changes are known among raptors between periods of productivity (Pavey and Nano 2013). Because small mammals, namely rodents, respond rapidly to rainfall‐mediated boom periods in our study area (Dickman, Mahon, et al. 1999; Letnic and Dickman 2010), particularly the long‐haired rat ( *Rattus villosissimus* ) (Greenville et al. 2013; Predavec and Dickman 1994), we also assessed how small mammals interacted with raptors between boom and bust periods. Our prediction was that small mammals would feature more in the diets of raptors in booms than busts, with the long‐haired rat dominating the assemblage of small mammals eaten whenever it was present (H3). Next, we expected that raptors exhibiting nomadic or locally nomadic movement patterns would have different dietary composition to sedentary species across boom and bust periods (H4), with sedentary species having more varied diets that were relatively invariant between productivity periods. Finally, we predicted that there should be no drift in dietary composition through time within groups of raptor species, with boom and bust dynamics instead being the primary driver of diet structuring (H5).

As climate change will likely exacerbate boom‐bust productivity dynamics (Hughes 2003), we postulate that this will affect prey abundance and community dynamics and in turn lead to restructuring of raptor diets via altered predator–prey interactions (Brown et al. 1997; Greenville et al. 2017). Understanding how the diets of raptors are currently structured permits us to infer how future change may influence raptor communities, allowing assessment of ongoing threats to these birds. Such research has global applications where climate change is expected to impact resource‐pulse environments. Although many threats to raptors are known (McClure et al. 2018), the potential impact from climate change is not well understood (Martínez‐Ruiz et al. 2023).

## 2 Material and Methods

### 2.1 Study Area and Data Collection

The study area is situated in the eastern Simpson Desert, approximately 120 km west of Bedourie, western Queensland. Work was conducted across Ethabuka and Cravens Peak cattle stations (now Ethabuka and Pilungah Reserves) and Carlo Station. Current and historic land use is a mix of grazing and nature reserve. The landscapes are characterised by long parallel sand dunes up to 1 km apart (Predavec and Dickman 1993). On these dunes, spinifex (*Triodia basedowii*) dominates, with patches of herbs and shrubs including species of *Acacia*, *Crotalaria*, *Grevillea*, *Dodonaea* and *Sida* (Wardle et al. 2015). In swales between dunes, hard claypans form and vegetation is typically woodland dominated by gidgee (*Acacia georginae*). Gibber habitats featuring stony plains with sparse vegetation occur sporadically, and patches of riparian vegetation dominated by coolabah (*Eucalyptus coolabah*) occur near isolated rivers and swamps. The desert has an average maximum temperature of 40°C in January (warmest month) and 23°C in June (coolest month). Average rainfall is low but highly variable (mean = 203.8 mm, 95% CI = 65.2–425.5 mm) (Bureau of Meteorology (BOM) 2025), with boom periods driven by episodic peak rainfall in summer followed by extended bust periods of very little rain.

We collated raptor dietary data by collecting regurgitated bird pellets and discarded prey remains opportunistically from confirmed roost or nesting trees for each species between June 1990 and October 2011. Analysing both pellets and prey remains minimises potential bias that may occur if only pellets or remains are analysed in isolation (Simmons et al. 1991). When possible, we cleared collection sites 2–3 months before pellets were collected for analysis to ensure they were fresh. Each pellet was broken open individually and the contents identified to the lowest taxonomic level possible, as per other studies (Dickman et al. 1991). For each pellet, we estimated the proportional volume occupied by each dietary component/prey item. This was preferred to other methods as it was easier to compare this metric between pellets of different species whilst providing an accurate indication of the dominant items within the respective diets (Dickman et al. 1991).

Whether pellets were recorded from a boom or bust period was determined by examining long‐term small mammal trapping records from within the study area and 12‐month preceding rainfall records from 12 independent weather stations setup across the region (Dickman et al. 2014); data were obtained from the closest weather station to pellet collection locations. Boom periods generally occurred 3–9 months after significant summer rainfall (> 200 mm) between December and February and were characterised by irruptions of small rodents such as the spinifex hopping‐mouse ( *Notomys alexis* ), sandy inland mouse ( *Pseudomys hermannsburgensis* ) and desert mouse ( *P. desertor* ). Irruptions of these species occurred in 1991, 1992, 1997, 1998, 2001, 2008 and 2011, with *R. villosissimus* also irrupting in 1991, 1992 and 2011 following extreme (95th quantile) summer rains (> 400 mm) (Dickman et al. 2014; Greenville et al. 2012, 2013). An irruption of small mammals in 1998 was unusual in that only 172 mm fell over the preceding summer, but wet conditions continued in winter (~40 mm) and rodent populations peaked 3 months later in October 1998 (Dickman et al. 2014). Raptor pellets collected outside these boom years coincided with dry periods and low rodent numbers and were designated as bust periods. This binary classification follows established understanding of how rainfall influences rodent dynamics in arid environments (Letnic et al. 2005) as well as the dynamics of other raptor prey taxa such as small insectivorous and granivorous birds (Tischler et al. 2013).

Animal ethics approval was provided by the University of Sydney Animal Ethics Committee, project numbers: L04/9‐94/3/1054, L04/5‐96/2/2361, L04/1‐98/3/2656, L04/4‐2000/1/3/3130, L04/2‐2001/3/3344, L04/4‐2004/3/3896, L04/1‐2007/3/4510, L04/4‐2009/3/5020, L04/4‐2010/3/5297, 2016/966, 2019/1521 and 2022/2100; and scientific permits for fieldwork from licensing authorities: T1182, T510, T737, T789, T1114, W0/000738/95/SAA, WO/000738/00/SAA, WISP02994105, WISP07623410, WITK07635510, WISP15192414, WITK15192514, WISP15192514, WA0006737, WA0052429 and P‐PTUKI‐100480312.

### 2.2 Statistical Methods

We created a matrix of dietary components from each pellet of every raptor species. All terrestrial mammals were identified to species, where feasible, or genus level. Due to difficulty in consistently identifying other remains to species or genus level, we placed birds, reptiles, amphibians, fish, bats (Microchiroptera) into their own groups, and plants and fungi into ‘other’. To address hypothesis 1, we calculated dietary niche breadth using Levins' standardised index (Levins 1968) across the pellets of all raptor species, within both boom and bust periods. This provides a value between 0 (only a single dietary component eaten) and 1 (all dietary components eaten in equal volumes). Next, we constructed a generalised linear model (GLM) using package ‘*glmmTMB*’ (Brooks et al. 2017) (R Version 4.3.1) that considered niche breadth as a response and period (boom or bust) as a predictor. The GLM was fitted with a gamma distribution as data were skewed positively and all zero values were converted to 0.0001 to permit model computation. This contracted the lower bound of the dataset slightly, since gamma family models cannot handle perfect zero values, and was undertaken as per other studies (Kosmidis and Zeileis 2025; Lubbe et al. 2021). Then, we determined the degree of overlap in the diets of raptor species within boom and bust periods independently. Overlap was calculated with Pianka's index (Pianka 1974), which provides a value between 0 (no overlap) and 1 (complete overlap). Only species with pellets recorded in both periods were included in this analysis. Dietary overlap between boom and bust periods was compared using a Mantel test with a Spearman's rank correlation coefficient, since data were non‐normally distributed, with 999 permutations.

For hypothesis 2, mammal species were classified as either ‘small mammal’ or ‘large mammal’; small mammals, including bats, weighed < 5.5 kg and large mammals were ≥ 5.5 kg, in line with other studies (Hunter et al. 2018). We undertook a multivariate analysis of abundance using the package ‘*mvabund*’ (Wang et al. 2012), fitted with a negative binomial distribution, to assess how the interaction of period and raptor species group (falconiform, accipitriform and strigiform), with period and species group as main effects, influenced the matrix of dietary components. We undertook post hoc analysis of variance with Bonferroni adjusted *p*‐values to assess how respective variables influenced each component. Then, we undertook a multivariate analysis of abundance to determine how species (main effect) and the interaction of species and period influenced the proportions of specific items in species' diets. Again, we employed post hoc analysis of variance with Bonferroni adjusted *p*‐values.

To assess hypothesis 3, we focused on terrestrial species that were part of the small mammal group (Appendix: Table S1). We created a matrix of these mammals and again analysed how raptors interacted with these prey during boom and bust periods. Only raptors that had consumed these mammals were included. To assess how the assemblage of small mammals differed across period and raptor species, we carried out multivariate analysis of abundance to determine the effect of the interaction between period with species, alongside species and period as independent main effects, on the proportions of small mammals recorded in pellets. We conducted post hoc analysis of variance with Bonferroni adjusted *p*‐values on this model.

For hypothesis 4, we firstly assigned each raptor species as being either nomadic, locally nomadic, or resident based on our own observations (Table 1) and literature (Birdlife Australia 2023). From this, we assessed if there was a difference in the assemblage of items eaten across species with a multivariate analysis of abundance that considered species movement status (nomadic, locally nomadic and resident) as a predictor alongside the interaction of species status and period, followed by a post hoc analysis of variance with Bonferroni correction, as before.

**TABLE 1:** Classification of raptor movement ecology based on existing literature (Birdlife Australia 2023) and our on‐site observations alongside totals of pellets recorded across years of survey.

Raptor species group	Species	Raptor species movement status	Year	Count
Accipitriform	Black kite	Locally nomadic	2004	22
			2009	19
			2011	14
	Black‐breasted buzzard	Locally nomadic	1992	30
			1994	37
			1996	34
			1997	12
	Black‐shouldered kite	Locally nomadic	1990	10
			1991	56
			1992	72
			1993	43
	Brown goshawk	Locally nomadic	1995	19
			1996	7
			1997	49
	Collared sparrowhawk	Locally nomadic	1996	15
			1998	15
			2005	9
	Letter‐winged kite	Nomadic	1991	62
			1993	75
	Little eagle	Nomadic	1995	44
	Spotted harrier	Locally nomadic	1991	13
			1992	8
			1997	8
			1998	16
	Square‐tailed kite	Nomadic	1992	59
	Swamp harrier	Nomadic	1994	14
			1996	4
			1997	11
			2007	4
	Wedge‐tailed eagle	Resident	1991	33
			1994	13
			1996	29
	Whistling kite	Locally nomadic	1991	15
			1994	19
Falconiform	Australian hobby	Locally nomadic	1997	34
			2001	17
	Black falcon	Nomadic	1991	47
			1995	9
			1996	12
	Brown falcon	Resident	1991	86
			1996	34
			1998	38
	Grey falcon	Locally nomadic	2009	30
			2011	41
	Nankeen kestrel	Resident	1995	60
			1997	81
	Peregrine falcon	Locally nomadic	1996	5
			1997	14
Strigiform	Barking owl	Resident	1992	27
			1994	19

For hypothesis 5, we calculated the average proportion of each diet component eaten by raptors within every year of sampling. We modelled a generalised additive model (GAM) using package *mgcv* (Pedersen et al. 2019), fitting year as a smoothed term interacted with diet component and the response variable being the average proportion of each diet component eaten. Smooths were fitted with thin‐plate regression splines. GAMs were constructed similarly across the raptor species groups and raptor movement status levels; however, we omitted the strigiform group from analyses due to lacking sufficient data. From here, we replaced zero values with 0.0001 to fit a beta distribution in the models, as before (Kosmidis and Zeileis 2025). We checked relevant assumptions for all models and GLMs were validated with package ‘*performance*’ (Lüdecke et al. 2021).

## 3 Results

Dietary data were obtained for 19 raptor species (Table S2), representing virtually all the raptors that are known to occur within the study region (Birdlife Australia 2023; Dickman et al. 2025). In total, 1444 pellets and prey remains were recorded and analysed (*n* = 742 in boom and 702 in bust periods). Dietary data for 17 raptors were recorded in both boom and bust periods. By pellets/prey remains, the most common raptors recorded were the black‐shouldered kite ( *Elanus axillaris* ) (*n* = 181, 12.5% of pellets) followed by the brown falcon ( *Falco berigora* ) (*n* = 158, 11%), nankeen kestrel ( *Falco cenchroides* ) (*n* = 141, 9.8%), letter‐winged kite (*n* = 137, 9.5%) and black‐breasted buzzard ( *Hamirostra melanosternon* ) (*n* = 113, 8%). Two species featured rarely, one each in only boom (square‐tailed kite ( *Lophoictinia isura* )) or bust (little eagle [ *Hieraaetus morphnoides* ]) periods.

### 3.1 Hypothesis 1

**FIGURE 2:**
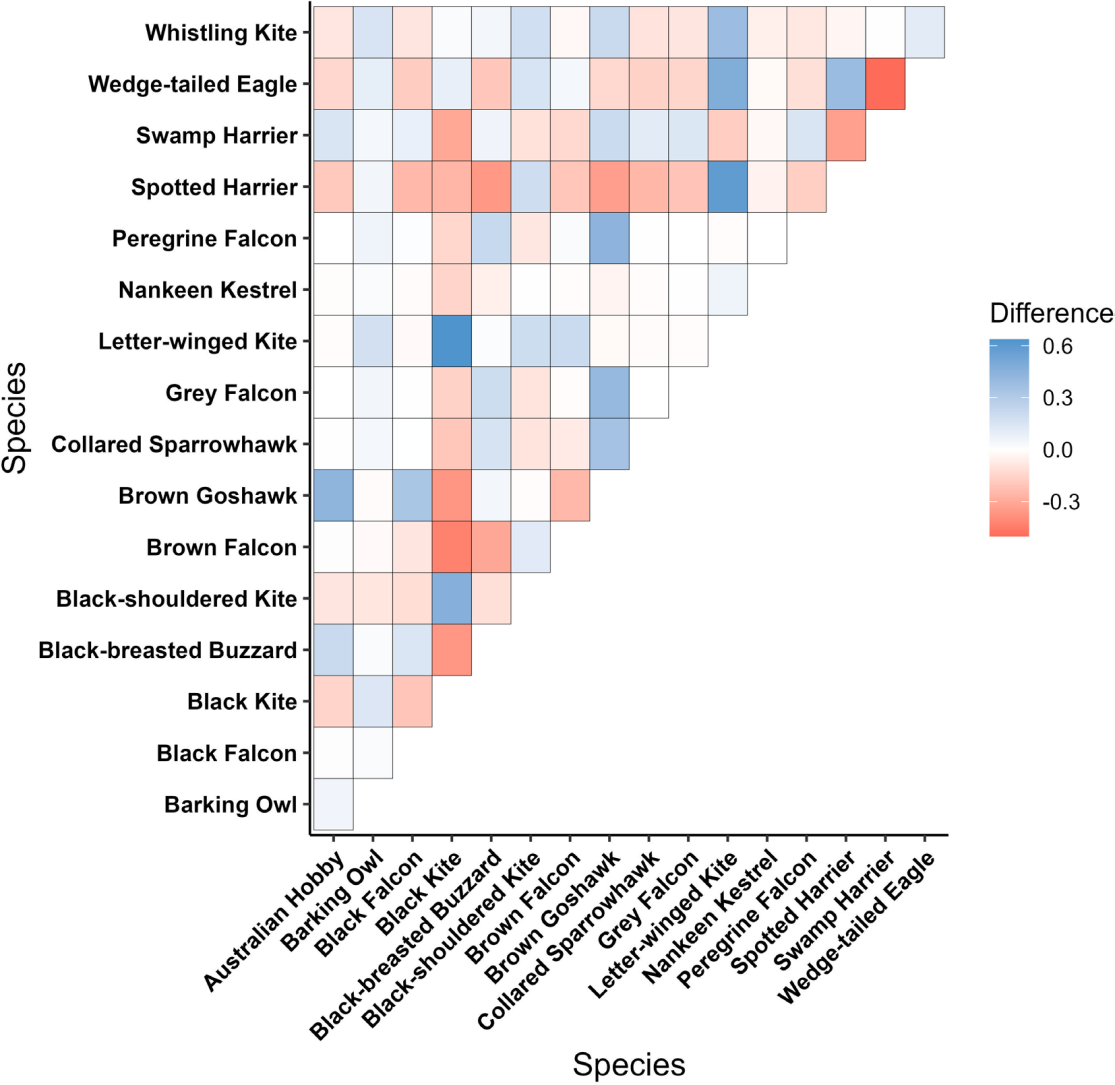
Differences in dietary overlap (Pianka's index) between all raptor species recorded in both boom and bust periods. Higher values indicate greater overlap in boom than bust periods.

Dietary niche breadth across all raptors was higher in bust compared to boom periods (*β* = 0.350, SE = 0.093, *Z* = 3.750, *p* < 0.001) (Figure S1). Within the assemblage of species recorded in both boom and bust periods, we found a strong positive relationship for inter‐specific dietary overlap (*r* = 0.855, *p* = 0.001); that is, species sharing high dietary overlap in bust periods continued to have high, or higher, overlap during booms while species with lower overlap did not differ significantly. Nonetheless, there was considerable variability in overlap between the diets of raptors (Figure 2), with species exhibiting differences between periods. The strongest changes in dietary overlap were noted for the letter‐winged kite, which showed increased overlap during boom periods with several species bar the swamp harrier ( *Circus approximans* ). Conversely, the black kite ( *Milvus migrans* ) and spotted harrier ( *Circus assimilis* ) showed marked decreases in dietary overlap with most species during booms, except the letter‐winged kite and black‐shouldered kite. Weaker changes in dietary overlap with other species were noted for the barking owl ( *Ninox connivens* ); these changes were largely positive or neutral. The brown goshawk ( *Accipiter fasciatus* ) also showed stronger positive overlap in diet during boom periods with several species of falcon as well as collared sparrowhawk ( *Accipiter cirrocephalus* ), swamp harrier and whistling kite ( *Haliastur sphenurus* ), although dietary overlap declined more markedly with the spotted harrier and wedge‐tailed eagle ( *Aquila audax* ).

### 3.2 Hypothesis 2

**FIGURE 3:**
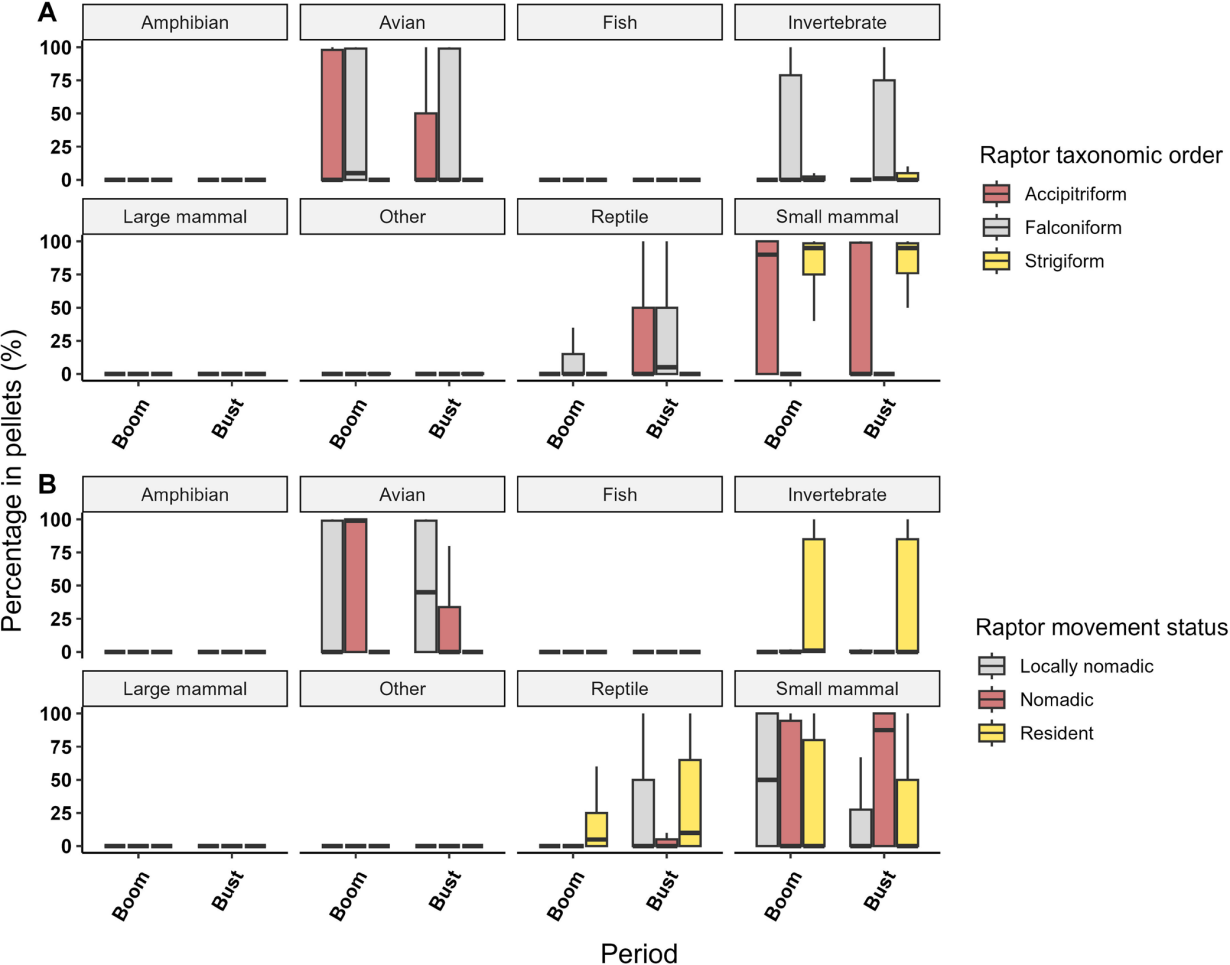
(A) Boxplots showing the proportions of different prey components in the diets of each raptor species group (taxonomic order) between boom and bust periods. (B) Boxplots showing the proportions of different prey components in the diets of raptors classified by movement status between boom and bust periods.

There was a strong interaction effect between species group and period on the proportions of dietary components represented in pellets (deviance = 31.7, *p* = 0.001), although the main effect of species group explained substantial deviance (Table S3). Post hoc tests indicated that this was driven by several dietary components (Table S3). Small mammals were eaten consistently and in high proportions by strigiforms (Figure 3A) across periods. Invertebrates were also taken but formed a lower proportion of the diet. Similarly, accipiters ate small mammals consistently between periods, but this varied more than for strigiforms, with small mammals forming 0%–100% of diet content between accipiter species (Figure 3A). The proportion of reptiles in the diets of accipiters declined markedly in the boom compared to bust. Conversely, birds formed a higher percentage of overall diet in booms. Birds and invertebrates were eaten consistently by falconiforms in both periods. However, reptiles were also eaten in considerably higher proportions in the bust compared to boom. Other items were eaten less frequently by all three species groups and did not dominate dietary intake.

**FIGURE 4:**
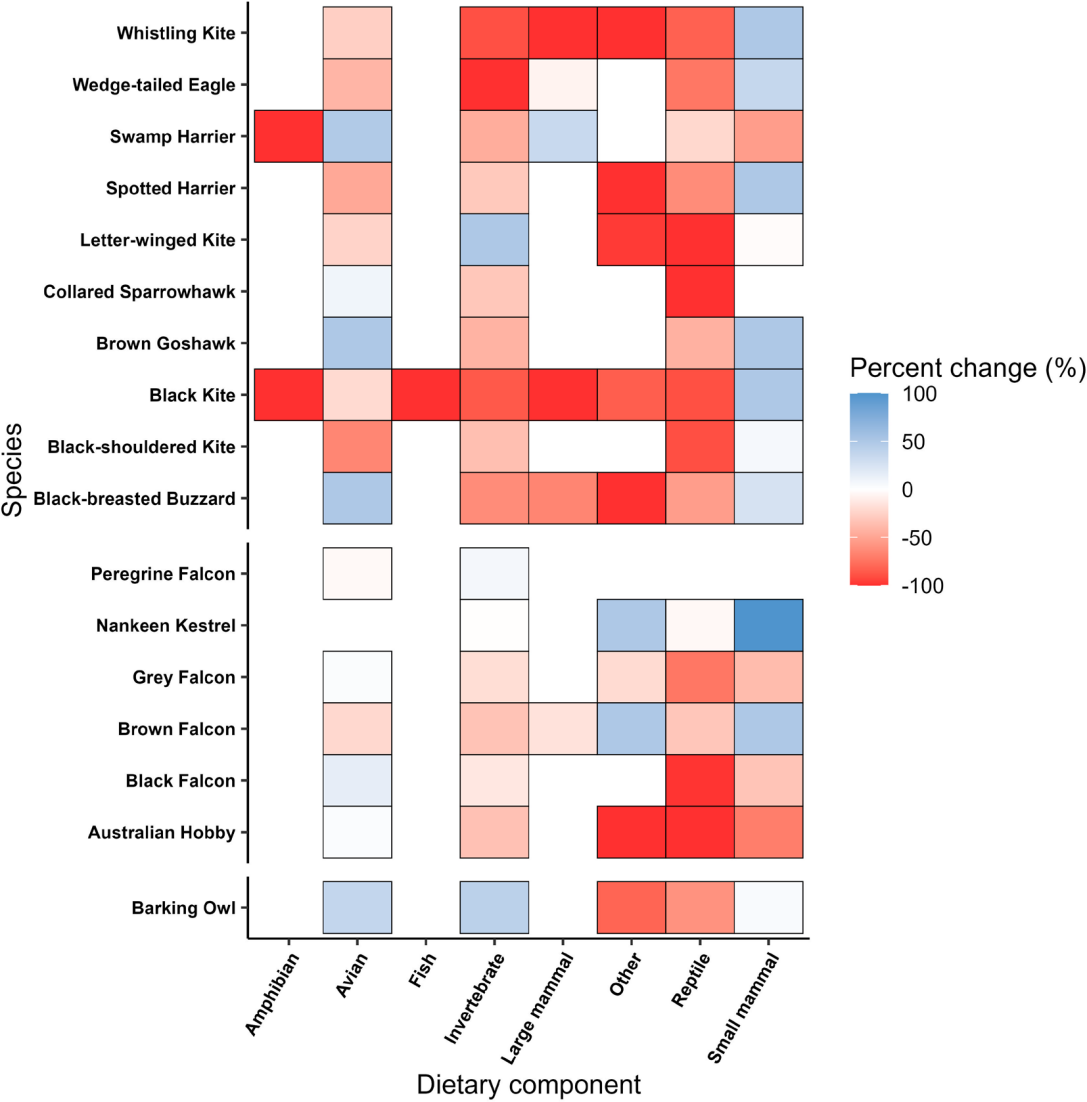
Differences in average proportions of each dietary component within the diets of raptor species across boom and bust periods. Higher percentage changes indicate greater proportions in the diet during booms. Only raptors that occurred in both boom and bust periods are included. Gaps indicate where a specific dietary item was not eaten by a raptor.

We found an interaction effect between species and period on dietary composition (deviance = 343.300, *p* = 0.001), although differences between species also explained significant deviance (Table S4). This was driven largely by small mammals, reptiles and invertebrates (Table S4). Of the prominent prey types (Table S2), reptiles were eaten less during booms across all species (Figure 4) while small mammals were generally eaten more, most notably by accipiters (Figure 4). Large mammals were generally less frequent components, as were invertebrates, across raptor species. The proportions of birds in the diets of falconiform species were relatively consistent but differed variably among strigiform and accipiter species.

### 3.3 Hypothesis 3

**FIGURE 5:**
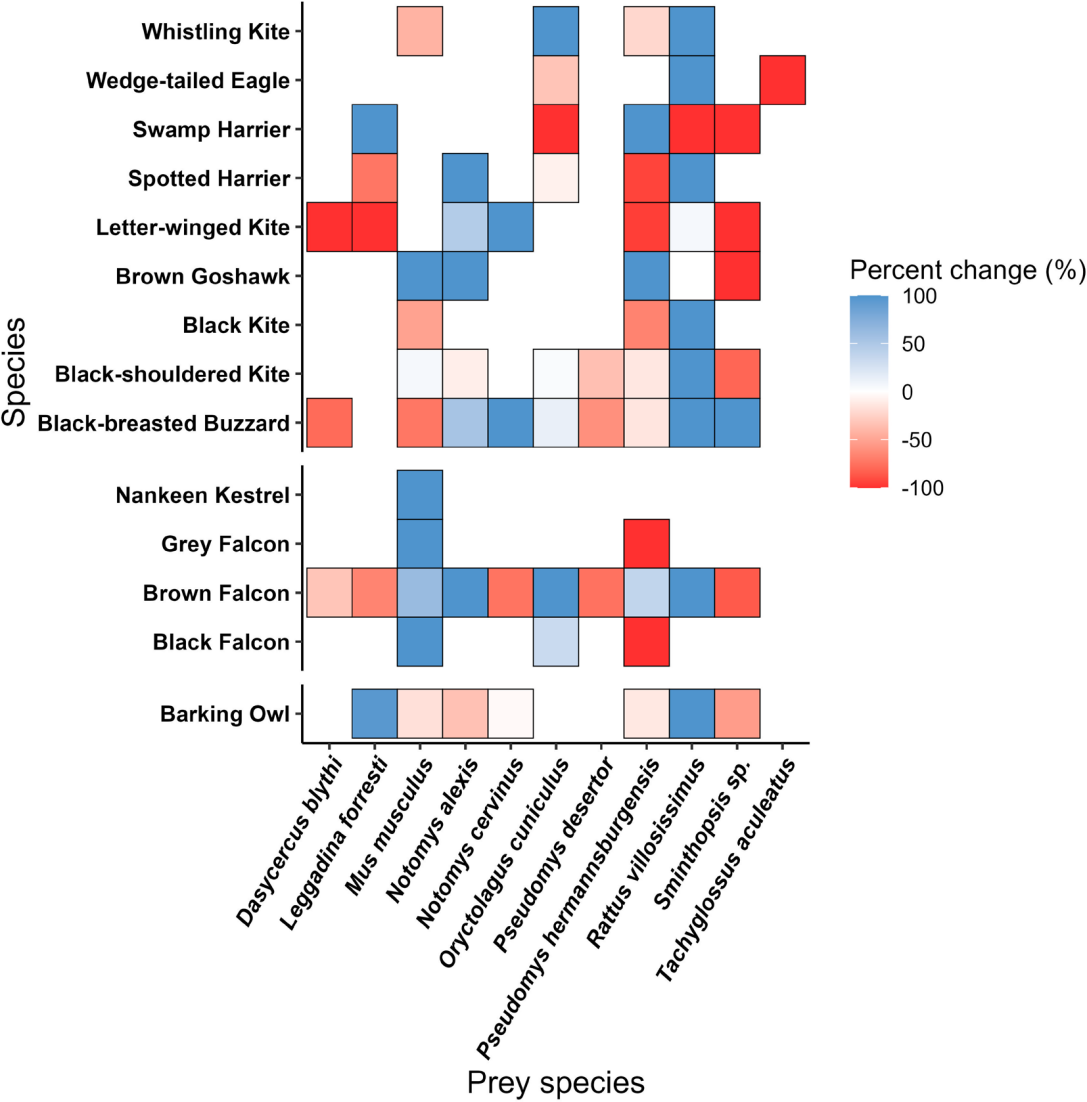
Differences in average proportions of each terrestrial small mammal species (< 5.5 kg) recorded in the diets of raptor species across boom and bust periods. Higher percentage changes indicate greater proportions in the diet during boom periods. Only raptors that fed on small mammals and occurred in both boom and bust periods are included. Gaps indicate where a specific small mammal species was not eaten by a raptor. Australian hobby ( *Falco longipennis* ) is not depicted as this raptor ate small mammals (see Figure 4) but in both instances these were Microchiroptera and not small terrestrial mammals.

There was an interaction effect between period and species on the proportions of small mammals consumed by raptors (deviance = 101.3, *p* = 0.011). This was driven by several species (Table S5), but was strongest for long‐haired rats (deviance = 45.406, *p* = 0.010). Consumption of the long‐haired rat increased in boom periods, with the notable exception of the swamp harrier (Figure 5). Other small mammals were eaten variably in boom and bust periods, but both the house mouse ( *Mus musculus* ) and spinifex hopping mouse ( *Notomys alexis* ) featured more in the diets of several raptors during booms. Again, species‐specific differences explained significant deviance in the data (Table S5).

### 3.4 Hypothesis 4

The representation of dietary components differed across the interaction of species movement status and period (deviance = 176.6, *p* = 0.001) (Table S6), with some of the deviance in the data also explained by species movement status in isolation (Table S6). Resident raptors ate invertebrates, small mammals, and, to a lesser extent, reptiles relatively consistently between booms and busts (Figure 3B). Nomadic species consistently ate small mammals but increased consumption of birds in boom periods. Locally nomadic species had variable diets, eating more reptiles than small mammals and a high proportion of birds in bust periods. However, during boom periods, they shifted their diets to more closely match nomadic species, mostly eating small mammals and birds (Figure 3B).

### 3.5 Hypothesis 5

**FIGURE 6:**
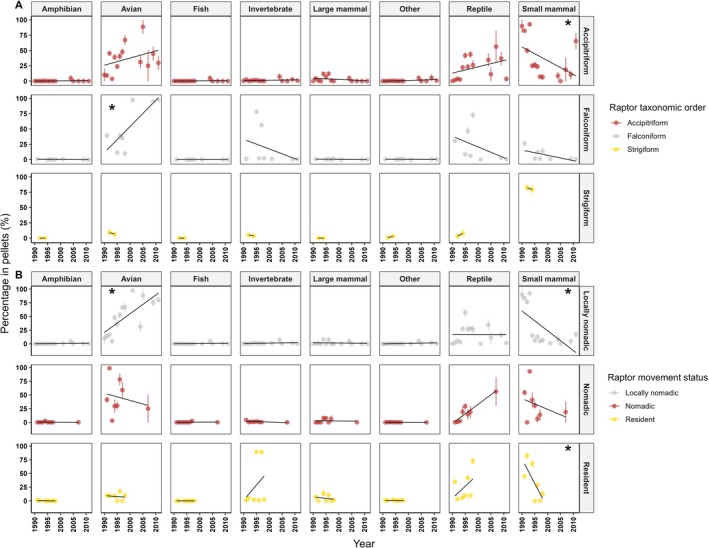
(A) Mean proportions of different prey components in the diets of different raptor species groups, plotted across year of sampling. (B) Mean proportions of different prey components in the diets of raptors with different movement ecology, plotted across year of sampling. Asterisks indicate relationships that show a statistical effect at *α* = 0.05.

The mean proportion of small mammals in the diets of accipitriform raptors declined across the sampling period (edf = 1.881, *χ* ^2^ = 15.337, *p* < 0.001; partial effect = 2.272 ± 0.563 [SE] in 1990 and partial effect = −0.433 ± 0.689 in 2011; Figure 6A). Birds increased in the diets of falconiforms over the same period (edf = 1.663, *χ* ^2^ = 14.335, *p* = 0.001; partial effect = −2.357 ± 0.773 in 1991 and partial effect = 2.457 ± 0.770 in 2011; Figure 6A). No other diet components were correlated with significant shifts temporally across any raptor species group (Table S7).

Locally nomadic raptors exhibited much greater feeding on birds through time (edf = 1.726, *χ* ^2^ = 13.776, *p* = 0.003), with partial effects increasing from −1.673 ± 0.523 in 1990 to 1.415 ± 0.759 in 2011 (Figure 6B). Conversely, over the same period, the proportions of small mammals in the diet of these raptors declined (edf = 1.795, *χ* ^2^ = 13.092, *p* = 0.001; Figure 6B), with partial effects decreasing from 2.036 ± 0.551 in 1990 to −1.063 ± 0.176 in 2011. Similarly, small mammals declined in the diets of resident raptors (edf = 1.000, *χ* ^2^ = 4.323, *p* = 0.038; Figure 6B), partial effects declined from 1.354 ± 0.651 in 1990 to −1.197 ± 0.576 in 1990. There was no significant correlation between year and average proportion of any items in the diets of nomadic raptors (Table S8).

## 4 Discussion

For the first time, we link boom‐bust dynamics in an arid environment to the dietary structuring of a species‐rich community of raptors. We found support for H1, with raptor dietary niche breadth decreasing in boom periods and expanding during busts. We also found that species sharing high dietary overlap in bust periods increased this overlap during booms. Nevertheless, at the community level, there were some differences between species. We found support for H2, with species groups and individual raptor species having distinct diets, but dietary structure nonetheless changed between boom and bust periods. Some support for H3 was evidenced as small mammals, notably the long‐haired rat, were eaten more in boom than in bust periods. However, this was not a consistent finding, as some raptors diverted feeding from small mammals to other groups during boom periods. We found further that resident species had varied diets that were relatively consistent through boom and bust periods, whereas the diets of nomadic and locally nomadic raptors fluctuated between periods, supporting H4. Contrastingly, hypothesis 5 was refuted. We found a dietary drift over time in some species groups and raptors with different movement ecology, with small mammals being eaten less and avian prey eaten more across the monitoring period.

### 4.1 Influence of Boom‐Bust Conditions on the Temporal Dynamics of Raptor Diets and Prey

Large rainfall events in the study area, as in arid environments elsewhere, exert significant, cascading effects through the ecosystem by increasing productivity and triggering population increases in primary consumers (Dickman et al. 2014). Resultantly, as most prey types are then abundant, raptors may forage more selectively for favoured prey, with decreased dietary (or niche) partitioning. This pattern was seen in our results, which indicated that overlap between species was positively correlated in boom compared to bust periods. Indeed, among raptors that favoured eating small mammals or birds in bust periods, there appeared to be higher overlap with other species which similarly shifted their diets in booms (Figure 2). Similar patterns of high overlap in diet within raptor assemblages when food is not limited, and dietary expansion when main prey become scarce, have been reported elsewhere (Jaksić and Braker 1983; Steenhof and Kochert 1988). Despite finding increased dietary overlap during boom times, however, shifts in diet during these periods still occurred. Thus, some species‐specific differences in diet were evident, mainly between raptors that favoured different prey types; for instance, falcons that favoured birds diverged from generalist species like the spotted harrier that shifted towards small mammals (Figures 2 and 4). Such shifts in partitioning may be important for promoting coexistence between species and for shaping the structure of raptor communities (Sarà et al. 2016; Ward et al. 2002).

The movement ecology of raptors also interacted with boom‐bust dynamics. Resident raptors exhibited diets that were relatively stable across booms and busts (Figure 3), reflecting their ability to forage on diverse prey that persist even during periods of low productivity. Conversely, nomadic and locally nomadic species exhibited significant shifts towards core dietary items (birds and small mammals) in boom periods, with this trend especially obvious for locally nomadic species (Figure 3). Nomadic species often have specialised diets regardless of background conditions, such as the letter‐winged kite in our study area (Lee et al. 2021). Despite showing some ability to feed on non‐preferred prey types (Figures 3, 4, 5), these raptors will not remain in a single location for an extended period due to insufficient availability of their core prey species over longer timeframes (Gangoso et al. 2013). Indeed, we saw the specialist letter‐winged kite exhibit little change in uptake of small mammals between periods (Figure 4). Therefore, boom periods may act as important facilitators of peak activity for these birds, in turn facilitating vital activities such as breeding (Pavey et al. 2020). Locally nomadic species like the grey falcon ( *Falco hypoleucos* ) may forage more broadly in bust periods (Figures 3 and 4) but then shift strongly towards preferred prey when conditions are favourable. Again, these species will move more widely but may not have to move as far as purely nomadic raptors since they exhibit, to some extent, greater dietary flexibility.

Of the small mammals that were identified in the diets of raptors, especially in boom conditions, long‐haired rats predominated (Figure 5). This species is highly irruptive following heavy summer rainfall events and occupies all desert habitats when its numbers peak (Greenville et al. 2013). Other rodents that respond positively to rainfall, such as the house mouse and spinifex hopping‐mouse (Brown and Singleton 1999; Greenville et al. 2016a), were also eaten more frequently by several raptor species during boom periods (Figure 5). Small mammals may be energetically and nutritionally preferable for many raptors (Fargallo et al. 2020), explaining why they were targeted more during boom periods when abundant (Figures 4 and 5). Comparatively, reptiles were generally eaten more in bust periods by raptors (Figures 3 and 4) when small mammals were scarce. Reptiles exhibit less response to rainfall than mammals (Greenville et al. 2016b; Read et al. 2012) and show higher abundance during bust periods than small mammals, thus allowing more opportunities for predation by raptors. Nonetheless, some species targeted reptiles. For example, brown falcons preyed upon gravid female thorny devils ( *Moloch horridus* ) to eat their eggs, with a pair recorded killing approximately 40 thorny devils during one breeding season. However, some reptiles also present as risky prey; for example, predation of the highly venomous western brown snake ( *Pseudonaja mengdeni* ) by brown falcons was observed only during bust periods. As avian predators often recognise and avoid dangerous prey (Brodie III 1993), reptiles may provide an important alternative prey source when conditions are harsh but are not favoured more broadly across the raptor community when alternative prey is abundant. This is likely to have important implications for reptiles, with boom periods potentially alleviating predation pressures exerted by raptors.

Temporal effects were also a prominent influence on the dietary composition of the arid raptor community, revealing that other factors also drive the observed trends. Interestingly, we found that small mammals formed a lesser proportion of the diets of accipiters, resident and locally nomadic raptors, whereas birds were a larger component of the diets of falconiforms and locally nomadic raptors across the monitoring period. Decline of small mammals in the diets of resident and locally nomadic raptors was surprising, as rodent irruptions are expected to increase in magnitude within the study area in the future (Greenville et al. 2012). Analysis of long‐term (100‐year) rainfall data indicates that exceptional rainfall events (95% quantile) have become larger in the Simpson Desert (Greenville et al. 2012), which was supported by our data which indicated an increase in the magnitude of 95% quantile rainfall events over the monitoring period (Figure S2). However, whilst 75% quantile rainfall remained stable, median (50% quantile), 25% and 5% quantile rainfall all declined in the same timeframe (Figure S2). Rainfall is naturally variable in the study area, and arid bioregions more broadly (Letnic and Dickman 2010), but localised decreases in rainfall during driest years and droughts are expected in the study region (Greenville et al. 2012). Small mammals typically respond negatively to low rainfall, although there are other exogenous factors like the abundance of predators that affect this (Letnic et al. 2011). Nonetheless, drought may outweigh these biotic factors, resulting in extremely low small mammal densities due to declines in available resources and changes in habitat utilisation (Dickman et al. 2011; Fox 2011).

Potentially, increasing variability in rainfall may be having flow‐on impacts to small mammal populations (Ventura‐Rojas et al. 2025), in turn affecting the foraging behaviour of raptors. Whilst some small mammals within the study area show asynchronous responses to rainfall (Greenville et al. 2016a), the core small mammal species recorded in the diets (Tables S1 and S5) are those that correlate most strongly with precipitation (Dickman, Mahon, et al. 1999; Greenville et al. 2013, 2016a; Kelly et al. 2013). Supporting this, only nomadic raptors showed no temporal differences in the proportion of small mammals in their diets, since these raptors exhibited the greatest reliance on small mammalian prey regardless of environmental conditions (Figure 3B). Comparatively, increased feeding on birds may reflect the permanency of many species across dry and wet periods (Tischler et al. 2013). Whilst avian abundance spikes during boom periods, with an influx of nomadic species alongside greater resource availability supporting breeding events (Burbidge and Fuller 2007; Tischler et al. 2013), many species are able to remain sedentary and survive by inhabiting heterogeneous habitats and remaining near water holes or riparian zones (Pavey and Nano 2009; Smith 2015). Accordingly, some raptor species may capitalise on these more available resources as other prey items fluctuate. This has implications under a changing climate, with climatic extremes predicted to increase in arid regions globally, resulting in overall drier ecosystems (Allan and Douville 2024) and large impacts on biota.

### 4.2 Species Group Differences

As expected, we found differences in how the diets of raptor species were structured as a function of rainfall‐driven boom‐bust dynamics, which contributed to explaining the trends found. However, these trends were not consistent across each species. The sole strigiform represented, the barking owl ( *Ninox connivens* ), hunts a diversity of prey but typically favours mammals and insects (Birdlife Australia 2023). Our findings underscored this, with the diet of this owl being relatively invariant between boom and bust periods for these two prey groups (Figure 3A). For falconiforms, several species specialise in birds, such as the grey falcon, peregrine falcon ( *Falco peregrinus* ), Australian hobby ( *F. longipennis* ) and black falcon ( *F. subniger* ) (Debus et al. 2021; Olsen et al. 2008; Schoenjahn et al. 2022). Indeed, we found birds to dominate the diets of these raptors, particularly in boom periods (Figure 4, Table S2). Comparatively, brown falcons exhibit greater flexibility in prey selection, taking terrestrial prey and invertebrates more than other avian‐specialist falcons (McDonald et al. 2012), possibly because of differences in morphology (Hull 1991). Brown falcons in our study also exhibited dietary plasticity, preying mostly on reptiles but shifting towards small mammals in boom periods. Nankeen kestrel diets are usually dominated by insects alongside smaller prey like reptiles, as we also observed (Olsen et al. 1979; Pryor 2021). Despite boom conditions being linked to increased insect activity (Kwok et al. 2016), the diet of the kestrel was relatively stable between periods, although kestrels did eat more small mammals during booms (Figure 4).

Accipitriforms were the most diverse group. Among these were avian specialists such as the collared sparrowhawk ( *Accipiter cirrocephalus* ) and brown goshawk (Aumann 1988; Olsen et al. 2018). However, most accipiter species exhibited generalist diets, hunting varied prey (Table S2), with the swamp harrier ( *Circus approximans* ) and letter‐winged kite being obvious exceptions. Despite often generalising, the swamp harrier prefers avian prey (Baker‐Gabb 1984); birds were the most abundant item in the diets of swamp harriers we recorded (Table S2), especially during boom periods. Letter‐winged kites specialise in small mammalian prey, particularly irruptive and abundant species (Lee et al. 2021; Pavey et al. 2020) such as long‐haired rats (Pavey et al. 2008). Small mammals were prominent in the diets of these kites (Table S2), but other items were also readily taken, especially invertebrates. Nevertheless, there was an evident shift towards small mammalian prey during boom periods across the accipiters with generalist diets (Figure 4). Large mammals were also a feature in the diets of the largest raptors, like wedge‐tailed eagles and black kites ( *Milvus migrans* ), and were probably scavenged as carrion (Table S2). However, the proportion of large mammals in raptor diets declined overall during booms, indicative that carrion may be a supplementary resource when live prey are less abundant (Meisuria et al. 2024). Since larger food items may be harder to detect in pellets, as soft tissues are typically targeted by facultative scavenging birds (Slabe et al. 2020), this may also be under‐represented in our sampling, although it is known that raptors scavenge more in harsher conditions (Meisuria et al. 2024). Other items such as frogs, fish and fungi/organic matter were eaten infrequently by all raptors (Table S2), either opportunistically or accidentally.

### 4.3 Conservation Implications and Future Directions

We emphasise how important climatic drivers are in structuring raptor diets in arid environments, as for other predators (Yip et al. 2015), with boom‐bust dynamics being critical determinants of dietary structuring and partitioning. Meanwhile, our study indicates that temporal variability in diet structure is evident for some raptors. This has implications as the climate changes and communities of prey species transform (Hughes 2003). While the threat of climate change is not well understood for raptor communities of arid regions, or more broadly (Martínez‐Ruiz et al. 2023), we speculate that by influencing ecological processes and thus prey species, these birds may face emerging threats from the loss or restructuring of prey assemblages (Brown et al. 1997). As an example, small passerines are forecast to decline in arid regions of Australia due to climate change contributing to more extreme heat events and prolonged drought (Gardner et al. 2022), potentially having deleterious effects on both generalist and avian‐specialist raptors (Reid and Fleming 1992). While other prey species in the study system may be more resilient to such changes, like some small mammals (Greenville et al. 2016a), this is inconsistent globally. For instance, there is evidence that a threatened raptor from southern Africa which specialises on small mammals, the black harrier ( *Circus maurus* ), may be exposed to reduced abundance of primary prey due to hotter temperatures (Garcia‐Heras et al. 2017). Similarly, we also found decreased proportions of small mammals in the diets of some raptors.

It follows that climatic variability leading to extended periods of unfavourable conditions for prey items may require arid‐dwelling raptors to broaden their diets for longer periods, depending upon adequate alternative prey availability. In the face of this, many arid raptors may decline (Iknayan and Beissinger 2018). However, the threat is likely to be most acute for species exhibiting dietary specialisation, which typically lack the ability to readily switch prey. Despite many of these birds being nomadic and able to forage over large areas for desired food, like the letter‐winged kite in this study, such traits do not guarantee fitness if unfavourable ‘bust’ conditions persist. These trends are reflected elsewhere, such as population modelling of another arid raptor, the tawny eagle ( *Aquila rapax* ) from southern Africa, demonstrating a decline in abundance due to long‐term decreases in rainfall and increasing precipitation variability associated with climate change (Wichmann et al. 2003). Similarly, the rarity of the nomadic grey falcon in Australian deserts has been linked to its extremely narrow diet, placing it at further risk should preferred avian prey become scarce (Schoenjahn 2013; Schoenjahn et al. 2022).

Conservation practitioners worldwide need to consider the potential impact of climate change on raptors in more detail, particularly threatened species. Many diet‐specialist and highly nomadic species are already listed as threatened with extinction, like the letter‐winged kite and grey falcon in this study system. Therefore, their declines may be exacerbated. Transformations of boom‐bust dynamics are also likely to compound other threatening processes that climate change interacts with. As an example, wildfires in desert systems will be affected by changes in precipitation and temperature, having corresponding effects on communities of prey for raptors, like songbirds (Connell et al. 2022) and mammals (Letnic and Dickman 2006). Thus, it is necessary that we modify how we manage these raptors with appreciation of interactive threats posed by climate change; indeed, understanding such threats to raptors more broadly is urgently required (McClure et al. 2018).

As our sampling was opportunistic, we have insufficient data to consider the interaction of breeding and non‐breeding diets with boom‐bust dynamics. Raptor diets often change between breeding and non‐breeding times (Barrows 1987; Panter and Amar 2022), so reproduction likely interacts with differing levels of prey abundance across boom and bust periods. Nevertheless, we were able to sample the diets of birds that were nesting as well as from roosts with no sign of breeding activity, so we expect data to be representative of both breeding and non‐breeding raptors. Future research would still benefit from systematic surveys as described elsewhere (Lewis et al. 2004; Schoenefuss et al. 2024). Our study region also contains predatory raptors but no vultures, with Australia the only continent lacking these obligate scavengers (Newsome et al. 2024). Community‐wide studies elsewhere in systems with vultures are critical to inform how raptor assemblages more broadly are affected by boom‐bust dynamics. Whilst scavenging occurred among some species in this study (Table S2), carrion is likely to be a more crucial resource elsewhere in the presence of vultures. Carrion inputs differ seasonally and may interact with boom‐bust dynamics (Moleón et al. 2019) whilst facultatively scavenging raptors often feed on carcasses more during conditions that are resource‐depauperate (Meisuria et al. 2024) (Figure 4). Undertaking such research is valuable since vultures, particularly old world vultures, are among the most threatened avifauna globally (McClure et al. 2018) but play a crucial ecosystem service by removing carrion from the landscape.

## 5 Conclusion

We show that boom and bust conditions have important ramifications for raptor foraging behaviour and dietary structuring in a desert environment, with marked differences evident between boom and bust periods of productivity. The observed trends were influenced by species‐specific prey preferences alongside the movement biology of raptors. Based on these results, it is probable that increasing droughts and climatic variability associated with climate change will threaten raptors due to the restructuring of ecosystem dynamics and prey communities. We suspect that this threat is most acute for raptors that are dietary specialists since these birds show a reduced ability to switch prey and may suffer most from unfavourable conditions that negatively affect their preferred prey types.

## Author Contributions

**Rhys J. Cairncross:** conceptualization (equal), formal analysis (lead), methodology (equal), writing – original draft (lead), writing – review and editing (equal). **Christopher R. Dickman:** conceptualization (equal), data curation (lead), funding acquisition (lead), methodology (lead), writing – review and editing (equal).

## Funding

This work was supported by the Australian Research Council and Terrestrial Ecology Research Network.

## Conflicts of Interest

The authors declare no conflicts of interest.

## Supporting information


**Appendix S1:** ece373002‐sup‐0001‐AppendixS1.xlsx.

**Appendix S2:** ece373002‐sup‐0002‐AppendixS2.docx.

## Data Availability

Data supporting this article have been uploaded as Supporting Information S1.

## References

[ece373002-bib-0001] Allan, R. P. , and H. Douville . 2024. “An Even Drier Future for the Arid Lands.” Proceedings of the National Academy of Sciences 121, no. 2: e2320840121. 10.1073/pnas.2320840121. PMC10786295 38157450

[ece373002-bib-0002] Aumann, T. 1988. “The Diet of the Brown Goshawk, *Accipiter‐fasciatu*s, in Southeastern Australia.” Wildlife Research 15, no. 6: 587–594. 10.1071/wr9880587.

[ece373002-bib-0003] Aumann, T. 2001. “An Intraspecific and Interspecific Comparison of Raptor Diets in the South‐West of the Northern Territory, Australia.” Wildlife Research 28, no. 4: 379. 10.1071/WR99092.

[ece373002-bib-0004] Ayal, Y. 2007. “Trophic Structure and the Role of Predation in Shaping Hot Desert Communities.” Journal of Arid Environments 68, no. 2: 171–187. 10.1016/j.jaridenv.2006.05.013.

[ece373002-bib-0005] Baker‐Gabb, D. J. 1984. “The Feeding Ecology and Behaviour of Seven Species of Raptor Overwintering in Coastal Victoria.” Wildlife Research 11, no. 3: 517–532. 10.1071/wr9840517.

[ece373002-bib-0006] Barrows, C. W. 1987. “Diet Shifts in Breeding and Nonbreeding Spotted Owls.” Journal of Raptor Research 21, no. 3: 95–97.

[ece373002-bib-0007] Bennison, K. , R. Godfree , and C. R. Dickman . 2018. “Synchronous Boom–Bust Cycles in Central Australian Rodents and Marsupials in Response to Rainfall and Fire.” Journal of Mammalogy 99, no. 5: 1137–1148. 10.1093/jmammal/gyy105.

[ece373002-bib-0008] Birdlife Australia . 2023. “Handbook of Australian, New Zealand & Antarctic Birds.” https://hanzab.birdlife.org.au/.

[ece373002-bib-0009] Brodie, E. D., III . 1993. “Differential Avoidance of Coral Snake Banded Patterns by Free‐Ranging Avian Predators in Costa Rica.” Evolution 47, no. 1: 227–235. 10.1111/j.1558-5646.1993.tb01212.x. 28568087

[ece373002-bib-0010] Brooks, M. E. , K. Kristensen , K. J. van Benthem , et al. 2017. “glmmTMB Balances Speed and Flexibility Among Packages for Zero‐Inflated Generalized Linear Mixed Modeling.” R Journal 9, no. 2: 378–400. 10.3929/ethz-b-000240890.

[ece373002-bib-0011] Brown, J. H. , T. J. Valone , and C. G. Curtin . 1997. “Reorganization of an Arid Ecosystem in Response to Recent Climate Change.” Proceedings of the National Academy of Sciences 94, no. 18: 9729–9733. 10.1073/pnas.94.18.9729. PMC23258 11038570

[ece373002-bib-0012] Brown, P. R. , and G. R. Singleton . 1999. “Rate of Increase as a Function of Rainfall for House Mouse *Mus domesticus* Populations in a Cereal‐Growing Region in Southern Australia.” Journal of Applied Ecology 36, no. 4: 484–493. 10.1046/j.1365-2664.1999.00422.x.

[ece373002-bib-0013] Buechley, E. R. , A. Santangeli , M. Girardello , et al. 2019. “Global Raptor Research and Conservation Priorities: Tropical Raptors Fall Prey to Knowledge Gaps.” Diversity and Distributions 25, no. 6: 856–869. 10.1111/ddi.12901.

[ece373002-bib-0014] Buij, R. , B. M. Croes , G. Gort , and J. Komdeur . 2013. “The Role of Breeding Range, Diet, Mobility and Body Size in Associations of Raptor Communities and Land‐Use in a West African Savanna.” Biological Conservation 166: 231–246. 10.1016/j.biocon.2013.06.028.

[ece373002-bib-0015] Burbidge, A. A. , and P. J. Fuller . 2007. “Gibson Desert Birds: Responses to Drought and Plenty.” Emu ‐ Austral Ornithology 107, no. 2: 126–134. 10.1071/MU06044.

[ece373002-bib-0016] Bureau of Meteorology (BOM) . 2025. “Climate Data Online [Dataset].” http://www.bom.gov.au/climate/data/.

[ece373002-bib-0017] Charter, M. , I. Izhaki , and A. Roulin . 2018. “The Relationship Between Intra–Guild Diet Overlap and Breeding in Owls in Israel.” Population Ecology 60, no. 4: 397–403. 10.1007/s10144-018-0633-6.

[ece373002-bib-0018] Chesson, P. , R. L. E. Gebauer , S. Schwinning , et al. 2004. “Resource Pulses, Species Interactions, and Diversity Maintenance in Arid and Semi‐Arid Environments.” Oecologia 141, no. 2: 236–253. 10.1007/s00442-004-1551-1. 15069635

[ece373002-bib-0019] Connell, J. , M. A. Hall , D. G. Nimmo , S. J. Watson , and M. F. Clarke . 2022. “Fire, Drought and Flooding Rains: The Effect of Climatic Extremes on Bird Species' Responses to Time Since Fire.” Diversity and Distributions 28, no. 3: 417–438. 10.1111/ddi.13287.

[ece373002-bib-0020] Debus, S. J. S. , T. S. Hatfield , G. S. Olde , and A. B. Rose . 2021. “Breeding Behaviour and Diet of a Pair of Black Falcons “*Falco subniger*” in Northern New South Wales.” Australian Field Ornithology 22, no. 4: 165–181. 10.3316/informit.345772066264869.

[ece373002-bib-0021] Dickman, C. R. , S. E. J. Daly , and G. W. Connell . 1991. “Dietary Relationships of the Barn Owl and Australian Kestrel on Islands Off the Coast of Western Australia.” Emu ‐ Austral Ornithology 91, no. 2: 69–72. 10.1071/MU9910069.

[ece373002-bib-0022] Dickman, C. R. , A. Greenville , G. M. Wardle , and C. R. Pavey . 2025. “Simpson Desert.” In Global Biome Conservation and Global Warming: Impacts on Ecology and Biodiversity, edited by G. L. Demolin‐Leite , P. Török , and J. C. B. Barrionuevo . Elsevier.

[ece373002-bib-0023] Dickman, C. R. , A. C. Greenville , B. Tamayo , and G. M. Wardle . 2011. “Spatial Dynamics of Small Mammals in Central Australian Desert Habitats: The Role of Drought Refugia.” Journal of Mammalogy 92, no. 6: 1193–1209. 10.1644/10-MAMM-S-329.1.

[ece373002-bib-0024] Dickman, C. R. , M. Letnic , and P. S. Mahon . 1999. “Population Dynamics of Two Species of Dragon Lizards in Arid Australia: The Effects of Rainfall.” Oecologia 119, no. 3: 357–366. 10.1007/s004420050796. 28307758

[ece373002-bib-0025] Dickman, C. R. , P. S. Mahon , P. Masters , and D. F. Gibson . 1999. “Long‐Term Dynamics of Rodent Populations in Arid Australia: The Influence of Rainfall.” Wildlife Research 26, no. 4: 389–403. 10.1071/wr97057.

[ece373002-bib-0026] Dickman, C. R. , G. M. Wardle , J. Foulkes , and N. de Preu . 2014. “Desert Complex Environments.” In Biodiversity and Environmental Change: Monitoring, Challenges and Direction, edited by E. Burns , D. Lindenmayer , A. Lowe , and N. Thurgate . CSIRO Publishing.

[ece373002-bib-0027] Fargallo, J. A. , J. Navarro‐López , P. Palma‐Granados , and R. M. Nieto . 2020. “Foraging Strategy of a Carnivorous‐Insectivorous Raptor Species Based on Prey Size, Capturability and Nutritional Components.” Scientific Reports 10, no. 1: 7583. 10.1038/s41598-020-64504-4. 32372048 PMC7200729

[ece373002-bib-0028] Fox, B. J. 2011. “Review of Small Mammal Trophic Structure in Drylands: Resource Availability, Use, and Disturbance.” Journal of Mammalogy 92, no. 6: 1179–1192. 10.1644/10-MAMM-S-227.1.

[ece373002-bib-0029] Foysal, M. , and C. T. Panter . 2024a. “Red‐Necked Falcons *Falco chicquera* Track Changing Environments by Shifting Their Trophic Niche During the Rainy Monsoon Season.” Bird Study 71, no. 1: 65–75. 10.1080/00063657.2024.2306510.

[ece373002-bib-0030] Foysal, M. , and C. T. Panter . 2024b. “Synergistic Effects of Climate and Urbanisation on the Diet of a Globally Near Threatened Subtropical Falcon.” Ecology and Evolution 14, no. 9: e70290. 10.1002/ece3.70290. 39257881 PMC11387113

[ece373002-bib-0031] Gangoso, L. , P. López‐López , J. M. Grande , et al. 2013. “Ecological Specialization to Fluctuating Resources Prevents Long‐Distance Migratory Raptors From Becoming Sedentary on Islands.” PLoS One 8, no. 4: e61615. 10.1371/journal.pone.0061615. 23626704 PMC3634022

[ece373002-bib-0032] Garcia‐Heras, M.‐S. , F. Mougeot , R. E. Simmons , and B. Arroyo . 2017. “Regional and Temporal Variation in Diet and Provisioning Rates Suggest Weather Limits Prey Availability for an Endangered Raptor.” Ibis 159, no. 3: 567–579. 10.1111/ibi.12478.

[ece373002-bib-0033] Gardner, J. L. , M. Clayton , R. Allen , J. Stein , and T. Bonnet . 2022. “The Effects of Temperature Extremes on Survival in Two Semi‐Arid Australian Bird Communities Over Three Decades, With Predictions to 2104.” Global Ecology and Biogeography 31, no. 12: 2498–2509. 10.1111/geb.13591.

[ece373002-bib-0034] Greenville, A. C. , G. M. Wardle , and C. R. Dickman . 2012. “Extreme Climatic Events Drive Mammal Irruptions: Regression Analysis of 100‐Year Trends in Desert Rainfall and Temperature.” Ecology and Evolution 2, no. 11: 2645–2658. 10.1002/ece3.377. 23170202 PMC3501619

[ece373002-bib-0035] Greenville, A. C. , G. M. Wardle , and C. R. Dickman . 2013. “Extreme Rainfall Events Predict Irruptions of Rat Plagues in Central Australia.” Austral Ecology 38, no. 7: 754–764. 10.1111/aec.12033.

[ece373002-bib-0036] Greenville, A. C. , G. M. Wardle , and C. R. Dickman . 2017. “Desert Mammal Populations Are Limited by Introduced Predators Rather Than Future Climate Change.” Royal Society Open Science 4, no. 11: 170384. 10.1098/rsos.170384. 29291051 PMC5717625

[ece373002-bib-0037] Greenville, A. C. , G. M. Wardle , V. Nguyen , and C. R. Dickman . 2016a. “Population Dynamics of Desert Mammals: Similarities and Contrasts Within a Multispecies Assemblage.” Ecosphere 7, no. 5: e01343. 10.1002/ecs2.1343.

[ece373002-bib-0038] Greenville, A. C. , G. M. Wardle , V. Nguyen , and C. R. Dickman . 2016b. “Spatial and Temporal Synchrony in Reptile Population Dynamics in Variable Environments.” Oecologia 182, no. 2: 475–485. 10.1007/s00442-016-3672-8. 27337964

[ece373002-bib-0039] Greenville, A. C. , G. M. Wardle , B. Tamayo , and C. R. Dickman . 2014. “Bottom‐Up and Top‐Down Processes Interact to Modify Intraguild Interactions in Resource‐Pulse Environments.” Oecologia 175, no. 4: 1349–1358. 10.1007/s00442-014-2977-8. 24908053

[ece373002-bib-0040] Heisler‐White, J. L. , A. K. Knapp , and E. F. Kelly . 2008. “Increasing Precipitation Event Size Increases Aboveground Net Primary Productivity in a Semi‐Arid Grassland.” Oecologia 158, no. 1: 129–140. 10.1007/s00442-008-1116-9. 18670792

[ece373002-bib-0041] Holmgren, M. , P. Stapp , C. R. Dickman , et al. 2006. “Extreme Climatic Events Shape Arid and Semiarid Ecosystems.” Frontiers in Ecology and the Environment 4, no. 2: 87–95. 10.1890/1540-9295(2006)004[0087:ECESAA]2.0.CO;2.

[ece373002-bib-0042] Hughes, L. 2003. “Climate Change and Australia: Trends, Projections and Impacts.” Austral Ecology 28, no. 4: 423–443. 10.1046/j.1442-9993.2003.01300.x.

[ece373002-bib-0043] Hull, C. 1991. “A Comparison of the Morphology of the Feeding Apparatus in the Peregrine Falcon, *Falco‐peregrinus*, and the Brown Falcon, *F‐berigora* (Falconiformes).” Australian Journal of Zoology 39, no. 1: 67–76. 10.1071/zo9910067.

[ece373002-bib-0044] Hunter, D. O. , M. Lagisz , V. Leo , S. Nakagawa , and M. Letnic . 2018. “Not All Predators Are Equal: A Continent‐Scale Analysis of the Effects of Predator Control on Australian Mammals.” Mammal Review 48, no. 2: 108–122. 10.1111/mam.12115.

[ece373002-bib-0045] Iknayan, K. J. , and S. R. Beissinger . 2018. “Collapse of a Desert Bird Community Over the Past Century Driven by Climate Change.” Proceedings of the National Academy of Sciences 115, no. 34: 8597–8602. 10.1073/pnas.1805123115. PMC6112692 30082401

[ece373002-bib-0046] Jaksic, F. M. 1985. “Toward Raptor Community Ecology: Behavior Bases of Assemblage Structure.” Journal of Raptor Research 19, no. 4: 107–112.

[ece373002-bib-0047] Jaksić, F. M. , and H. E. Braker . 1983. “Food‐Niche Relationships and Guild Structure of Diurnal Birds of Prey: Competition Versus Opportunism.” Canadian Journal of Zoology 61, no. 10: 2230–2241. 10.1139/z83-295.

[ece373002-bib-0048] Jordan, R. , A. I. James , D. Moore , and D. C. Franklin . 2017. “Boom and Bust (or Not?) Among Birds in an Australian Semi‐Desert.” Journal of Arid Environments 139: 58–66. 10.1016/j.jaridenv.2016.12.013.

[ece373002-bib-0049] Kelly, L. T. , R. Dayman , D. G. Nimmo , M. F. Clarke , and A. F. Bennett . 2013. “Spatial and Temporal Drivers of Small Mammal Distributions in a Semi‐Arid Environment: The Role of Rainfall, Vegetation and Life‐History.” Austral Ecology 38, no. 7: 786–797. 10.1111/aec.12018.

[ece373002-bib-0050] Kosmidis, I. , and A. Zeileis . 2025. “Extended‐Support Beta Regression for [0, 1] Responses.” Journal of the Royal Statistical Society. Series C, Applied Statistics 75: qlaf039. 10.1093/jrsssc/qlaf039.

[ece373002-bib-0051] Kwok, A. B. C. , G. M. Wardle , A. C. Greenville , and C. R. Dickman . 2016. “Long‐Term Patterns of Invertebrate Abundance and Relationships to Environmental Factors in Arid Australia.” Austral Ecology 41, no. 5: 480–491. 10.1111/aec.12334.

[ece373002-bib-0052] Lee, J. S. , M. Letnic , and C. H. Mills . 2021. “Diet and Occurrences of the Letter‐Winged Kite in a Predation Refuge.” Science of Nature 108, no. 6: 61. 10.1007/s00114-021-01772-8. 34797399

[ece373002-bib-0053] Letnic, M. , and C. R. Dickman . 2006. “Boom Means Bust: Interactions Between the El Niño/Southern Oscillation (ENSO), rainfall and the Processes Threatening Mammal Species in Arid Australia.” Biodiversity and Conservation 15, no. 12: 3847–3880. 10.1007/s10531-005-0601-2.

[ece373002-bib-0054] Letnic, M. , and C. R. Dickman . 2010. “Resource Pulses and Mammalian Dynamics: Conceptual Models for Hummock Grasslands and Other Australian Desert Habitats.” Biological Reviews 85, no. 3: 501–521. 10.1111/j.1469-185X.2009.00113.x. 20015313

[ece373002-bib-0055] Letnic, M. , P. Story , G. Story , J. Field , O. Brown , and C. R. Dickman . 2011. “Resource Pulses, Switching Trophic Control, and the Dynamics of Small Mammal Assemblages in Arid Australia.” Journal of Mammalogy 92, no. 6: 1210–1222. 10.1644/10-MAMM-S-229.1.

[ece373002-bib-0056] Letnic, M. , B. Tamayo , and C. R. Dickman . 2005. “The Responses of Mammals to La Niña (El Niño Southern Oscillation)–Associated Rainfall, Predation, and Wildfire in Central Australia.” Journal of Mammalogy 86, no. 4: 689–703. 10.1644/1545-1542(2005)086[0689:TROMTL]2.0.CO;2.

[ece373002-bib-0057] Levins, R. 1968. Evolution in Changing Environments: Some Theoretical Explorations. Princeton University Press.

[ece373002-bib-0058] Lewis, S. B. , M. R. Fuller , and K. Titus . 2004. “A Comparison of 3 Methods for Assessing Raptor Diet During the Breeding Season.” Wildlife Society Bulletin 32, no. 2: 373–385. 10.2193/0091-7648(2004)32[373:ACOMFA]2.0.CO;2.

[ece373002-bib-0059] Lubbe, S. , P. Filzmoser , and M. Templ . 2021. “Comparison of Zero Replacement Strategies for Compositional Data With Large Numbers of Zeros.” Chemometrics and Intelligent Laboratory Systems 210: 104248. 10.1016/j.chemolab.2021.104248.

[ece373002-bib-0060] Lüdecke, D. , M. S. Ben‐Shachar , I. Patil , P. Waggoner , and D. Makowski . 2021. “Performance: An R Package for Assessment, Comparison and Testing of Statistical Models.” Journal of Open Source Software 6, no. 60: 3139. 10.21105/joss.03139.

[ece373002-bib-0061] Martínez‐Ruiz, M. , C. R. Dykstra , T. L. Booms , and M. T. Henderson . 2023. “Conservation Letter: Effects of Global Climate Change on Raptors.” Journal of Raptor Research 57, no. 1: 92–105. 10.3356/JRR-22-75.

[ece373002-bib-0062] McClure, C. J. W. , J. R. S. Westrip , J. A. Johnson , et al. 2018. “State of the World's Raptors: Distributions, Threats, and Conservation Recommendations.” Biological Conservation 227: 390–402. 10.1016/j.biocon.2018.08.012.

[ece373002-bib-0063] McDonald, P. G. , J. Olsen , and A. B. Rose . 2012. “The Diet of Breeding Brown Falcons (*Falco berigora*) in the Canberra Region, Australia, With Comparisons to Other Regions.” Journal of Raptor Research 46, no. 4: 394–400. 10.3356/JRR-12-05.1.

[ece373002-bib-0064] Meisuria, N. , E. E. Spencer , R. J. Cairncross , M. S. Crowther , and T. M. Newsome . 2024. “Scavenging and Social Interaction of an Apex Avian Scavenger Is Governed by Bioregional and Seasonal Variation.” Oikos 2024, no. 10: e10826. 10.1111/oik.10826.

[ece373002-bib-0065] Moleón, M. , N. Selva , M. M. Quaggiotto , D. M. Bailey , A. Cortés‐Avizanda , and T. L. DeVault . 2019. “Carrion Availability in Space and Time.” In Carrion Ecology and Management, edited by P. P. Olea , P. Mateo‐Tomás , and J. A. Sánchez‐Zapata , 23–44. Springer International Publishing. 10.1007/978-3-030-16501-7_2.

[ece373002-bib-0066] Morton, S. 2022. Australian Deserts: Ecology and Landscapes. Csiro Publishing.

[ece373002-bib-0067] Newsome, T. M. , R. Cairncross , C. X. Cunningham , et al. 2024. “Scavenging With Invasive Species.” Biological Reviews 99, no. 2: 562–581. 10.1111/brv.13035. 38148253

[ece373002-bib-0068] Nielsen, J. M. , E. L. Clare , B. Hayden , M. T. Brett , and P. Kratina . 2018. “Diet Tracing in Ecology: Method Comparison and Selection.” Methods in Ecology and Evolution 9, no. 2: 278–291. 10.1111/2041-210X.12869.

[ece373002-bib-0069] O'Bryan, C. J. , Z. Xie , H. Li , et al. 2025. “Rapid Global Deforestation Leaves Forest‐Dependent Raptors With Half of Their Suitable Habitat Remaining.” Global Change Biology 31, no. 11: e70603. 10.1111/gcb.70603. 41255350 PMC12628116

[ece373002-bib-0070] Olsen, J. , E. Fuentes , D. M. Bird , A. B. Rose , and D. Judge . 2008. “Dietary Shifts Based Upon Prey Availability in Peregrine Falcons and Australian Hobbies Breeding Near Canberra, Australia.” Journal of Raptor Research 42, no. 2: 125–137. 10.3356/JRR-07-19.1.

[ece373002-bib-0071] Olsen, J. , D. Judge , S. Trost , and A. B. Rose . 2018. “Diets of Breeding Brown Goshawks Accipiter Fasciatus and Collared Sparrowhawks *A. Cirrocephalus* Near Canberra, Australia and Comparisons With Other Regions and Raptors.” Corella 42: 18–28.

[ece373002-bib-0072] Olsen, P. , W. J. M. Vestjens , and J. Olsen . 1979. “Observations on the Diet of the Australian Kestrel *Falco cenchroides* .” Emu ‐ Austral Ornithology 79, no. 3: 133–138. 10.1071/MU9790133.

[ece373002-bib-0073] Panter, C. T. , and A. Amar . 2022. “Using Web‐Sourced Photographs to Examine Temporal Patterns in Sex‐Specific Diet of a Highly Sexually Dimorphic Raptor.” Royal Society Open Science 9, no. 10: 220779. 10.1098/rsos.220779. 36300138 PMC9579756

[ece373002-bib-0074] Pastro, L. A. , C. R. Dickman , and M. Letnic . 2013. “Effects of Wildfire, Rainfall and Region on Desert Lizard Assemblages: The Importance of Multi‐Scale Processes.” Oecologia 173, no. 2: 603–614. 10.1007/s00442-013-2642-7. 23494288

[ece373002-bib-0075] Pavey, C. R. 2021. “A Nomadic Avian Predator Displays Flexibility in Prey Choice During Episodic Outbreaks of Rodents in Arid Australia.” Oecologia 196, no. 1: 211–222. 10.1007/s00442-021-04926-7. 33934187

[ece373002-bib-0076] Pavey, C. R. , J. Gorman , and M. Heywood . 2008. “Dietary Overlap Between the Nocturnal Letter‐Winged Kite *Elanus scriptus* and Barn Owl *Tyto alba* During a Rodent Outbreak in Arid Australia.” Journal of Arid Environments 72, no. 12: 2282–2286. 10.1016/j.jaridenv.2008.07.013.

[ece373002-bib-0077] Pavey, C. R. , and C. E. M. Nano . 2009. “Bird Assemblages of Arid Australia: Vegetation Patterns Have a Greater Effect Than Disturbance and Resource Pulses.” Journal of Arid Environments 73, no. 6: 634–642. 10.1016/j.jaridenv.2009.01.010.

[ece373002-bib-0078] Pavey, C. R. , and C. E. M. Nano . 2013. “Changes in Richness and Abundance of Rodents and Native Predators in Response to Extreme Rainfall in Arid Australia.” Austral Ecology 38, no. 7: 777–785. 10.1111/aec.12062.

[ece373002-bib-0079] Pavey, C. R. , L. M. Nunn , P. J. Nunn , and C. E. M. Nano . 2020. “Nine Months in the Simpson Desert: The Anatomy of a Letter‐Winged Kite Breeding Irruption.” Journal of Arid Environments 177: 104138. 10.1016/j.jaridenv.2020.104138.

[ece373002-bib-0080] Pedersen, E. J. , D. L. Miller , G. L. Simpson , and N. Ross . 2019. “Hierarchical Generalized Additive Models in Ecology: An Introduction With mgcv.” PeerJ 7: e6876. 10.7717/peerj.6876. 31179172 PMC6542350

[ece373002-bib-0081] Piana, R. P. , and S. J. Marsden . 2012. “Diversity, Community Structure, and Niche Characteristics Within a Diurnal Raptor Assemblage of Northwestern Peru.” Condor 114, no. 2: 279–289. 10.1525/cond.2012.100163.

[ece373002-bib-0082] Pianka, E. R. 1974. “Niche Overlap and Diffuse Competition.” Proceedings of the National Academy of Sciences 71, no. 5: 2141–2145. 10.1073/pnas.71.5.2141. PMC388403 4525324

[ece373002-bib-0083] Ponce, C. , F. S. Carevic , and E. R. Carmona . 2018. “Seasonal Diet by a Generalist Raptor: The Case of the Variable Hawk (*Geranoaetus polyosoma*) at Atacama Desert, Northern Chile.” New Zealand Journal of Zoology 45, no. 2: 171–179. 10.1080/03014223.2017.1395750.

[ece373002-bib-0084] Porter, C. K. , J. Golcher‐Benavides , and C. W. Benkman . 2022. “Seasonal Patterns of Dietary Partitioning in Vertebrates.” Ecology Letters 25, no. 11: 2463–2475. 10.1111/ele.14100. 36134722

[ece373002-bib-0085] Predavec, M. , and C. R. Dickman . 1993. “Ecology of Desert Frogs: A Study From Southwestern Queensland.” In Herpetology in Australia: A Diverse Discipline, edited by D. Lunney and D. Ayers . Royal Zoological Society of New South Wales. 10.7882/HIA.1993.

[ece373002-bib-0086] Predavec, M. , and C. R. Dickman . 1994. “Population Dynamics and Habitat Use of the Long‐Haired Rat (*Rattus villosissimus*) in South‐Western Queensland.” Wildlife Research 21, no. 1: 1–9. 10.1071/wr9940001.

[ece373002-bib-0087] Pryor, K. 2021. “Predation and Prey‐Caching of Eastern Water Skinks *Eulamprus quoyii* by Nesting Nankeen Kestrels *Falco cenchroides* in Eastern New South Wales.” Australian Field Ornithology 38: 193–200. 10.20938/afo38193200.

[ece373002-bib-0088] Read, J. L. , K.‐J. Kovac , B. W. Brook , and D. A. Fordham . 2012. “Booming During a Bust: Asynchronous Population Responses of Arid Zone Lizards to Climatic Variables.” Acta Oecologica 40: 51–61. 10.1016/j.actao.2011.09.006.

[ece373002-bib-0089] Reid, J. , and M. Fleming . 1992. “The Conservation Status of Birds in Arid Australia.” Rangeland Journal 14, no. 2: 65–91. 10.1071/rj9920065.

[ece373002-bib-0090] Robinson, S. K. 1994. “Habitat Selection and Foraging Ecology of Raptors in Amazonian Peru.” Biotropica 26, no. 4: 443–458.

[ece373002-bib-0091] Sarà, M. , R. Mascara , and P. López‐López . 2016. “Understanding the Coexistence of Competing Raptors by Markov Chain Analysis Enhances Conservation of Vulnerable Species.” Journal of Zoology 299, no. 3: 163–171. 10.1111/jzo.12340.

[ece373002-bib-0092] Schlesinger, C. A. , K. A. Christian , C. D. James , and S. R. Morton . 2010. “Seven Lizard Species and a Blind Snake: Activity, Body Condition and Growth of Desert Herpetofauna in Relation to Rainfall.” Australian Journal of Zoology 58, no. 5: 273–283. 10.1071/ZO10058.

[ece373002-bib-0093] Schoenefuss, P. , A. S. Kutt , P. L. Kern , et al. 2024. “An Investigation Into the Utility of Eastern Barn Owl Pellet Content as a Tool to Monitor Small Mammal Diversity in an Arid Ecosystem.” Austral Ecology 49, no. 3: e13503. 10.1111/aec.13503.

[ece373002-bib-0094] Schoenjahn, J. 2013. “A Hot Environment and One Type of Prey: Investigating Why the Grey Falcon (*Falco hypoleucos*) is Australia's Rarest Falcon.” Emu ‐ Austral Ornithology 113, no. 1: 19–25. 10.1071/MU12049.

[ece373002-bib-0095] Schoenjahn, J. , C. R. Pavey , and G. H. Walter . 2022. “Has the Australian Endemic Grey Falcon the Most Extreme Dietary Specialization Among All Falco Species?” Animals 12, no. 12: 12. 10.3390/ani12121582. PMC9219490 35739918

[ece373002-bib-0096] Seymour, C. L. , R. E. Simmons , G. S. Joseph , and J. A. Slingsby . 2015. “On Bird Functional Diversity: Species Richness and Functional Differentiation Show Contrasting Responses to Rainfall and Vegetation Structure in an Arid Landscape.” Ecosystems 18, no. 6: 971–984. 10.1007/s10021-015-9875-8.

[ece373002-bib-0097] Simmons, R. E. , D. M. Avery , and G. Avery . 1991. “Biases in Diets Determined From Pellets and Remains: Correction Factors for a Mammal and Bird‐Eating Raptor.” Journal of Raptor Research 25, no. 3: 63–67.

[ece373002-bib-0098] Slabe, V. A. , J. T. Anderson , J. Cooper , et al. 2020. “Feeding Ecology Drives Lead Exposure of Facultative and Obligate Avian Scavengers in the Eastern United States.” Environmental Toxicology and Chemistry 39, no. 4: 882–892. 10.1002/etc.4680. 32022303

[ece373002-bib-0099] Smith, J. E. 2015. “Effects of Environmental Variation on the Composition and Dynamics of an Arid‐Adapted Australian Bird Community.” Pacific Conservation Biology 21, no. 1: 74–86. 10.1071/PC14905.

[ece373002-bib-0100] Spencer, E. E. , T. M. Newsome , and C. R. Dickman . 2017. “Prey Selection and Dietary Flexibility of Three Species of Mammalian Predator During an Irruption of Non‐Cyclic Prey.” Royal Society Open Science 4, no. 9: 170317. 10.1098/rsos.170317. 28989739 PMC5627079

[ece373002-bib-0101] Steenhof, K. , and M. N. Kochert . 1988. “Dietary Responses of Three Raptor Species to Changing Prey Densities in a Natural Environment.” Journal of Animal Ecology 57, no. 1: 37–48. 10.2307/4761.

[ece373002-bib-0102] Tatler, J. , T. A. A. Prowse , D. A. Roshier , B. L. Allen , and P. Cassey . 2019. “Resource Pulses Affect Prey Selection and Reduce Dietary Diversity of Dingoes in Arid Australia.” Mammal Review 49, no. 3: 263–275. 10.1111/mam.12157.

[ece373002-bib-0103] Tischler, M. , C. R. Dickman , and G. M. Wardle . 2013. “Avian Functional Group Responses to Rainfall Across Four Vegetation Types in the Simpson Desert, Central Australia.” Austral Ecology 38, no. 7: 809–819. 10.1111/aec.12065.

[ece373002-bib-0104] Ventura‐Rojas, P. D. , A. González‐Romero , C. E. Moreno , and V. J. Sosa . 2025. “Effect of Rainfall, Temperature and Climate Change on the Ecology of the Rodents of Arid Zones: A Review.” Mammal Review 55, no. 2: e12372. 10.1111/mam.12372.

[ece373002-bib-0105] Vernes, K. , S. M. Jackson , T. F. Elliott , M. Tischler , and A. Harper . 2021. “Diets of Mammalian Carnivores in the Deserts of North‐Eastern South Australia.” Journal of Arid Environments 188: 104377. 10.1016/j.jaridenv.2020.104377.

[ece373002-bib-0106] Vidal‐Mateo, J. , J. Benavent‐Corai , P. López‐López , et al. 2022. “Search Foraging Strategies of Migratory Raptors Under Different Environmental Conditions.” Frontiers in Ecology and Evolution 10: 666238. 10.3389/fevo.2022.666238.

[ece373002-bib-0107] Wang, Y. , U. Naumann , S. T. Wright , and D. I. Warton . 2012. “Mvabund – An R Package for Model‐Based Analysis of Multivariate Abundance Data.” Methods in Ecology and Evolution 3, no. 3: 471–474. 10.1111/j.2041-210X.2012.00190.x.

[ece373002-bib-0108] Ward, A. B. , P. D. Weigl , and R. M. Conroy . 2002. “Functional Morphology of Raptor Hindlimbs: Implications for Resource Partitioning.” Auk 119, no. 4: 1052–1063. 10.1093/auk/119.4.1052.

[ece373002-bib-0109] Wardle, G. M. , A. C. Greenville , A. S. K. Frank , M. Tischler , N. J. Emery , and C. R. Dickman . 2015. “Ecosystem Risk Assessment of Georgina Gidgee Woodlands in Central Australia.” Austral Ecology 40, no. 4: 444–459. 10.1111/aec.12265.

[ece373002-bib-0110] Wardle, G. M. , C. R. Pavey , and C. R. Dickman . 2013. “Greening of Arid Australia: New Insights From Extreme Years.” Austral Ecology 38, no. 7: 731–740. 10.1111/aec.12073.

[ece373002-bib-0111] Wichmann, M. C. , F. Jeltsch , W. R. J. Dean , K. A. Moloney , and C. Wissel . 2003. “Implication of Climate Change for the Persistence of Raptors in Arid Savanna.” Oikos 102, no. 1: 186–202. 10.1034/j.1600-0706.2003.12044.x.

[ece373002-bib-0112] Yang, L. H. , K. F. Edwards , J. E. Byrnes , J. L. Bastow , A. N. Wright , and K. O. Spence . 2010. “A Meta‐Analysis of Resource Pulse–Consumer Interactions.” Ecological Monographs 80, no. 1: 125–151. 10.1890/08-1996.1.

[ece373002-bib-0113] Yip, S. J. S. , and C. R. Dickman . 2023. “Foraging and Food Selection in a Desert Rodent: Diet Shifts of the Sandy Inland Mouse Between Population Booms and Busts.” Animals 13, no. 10: 10. 10.3390/ani13101702. PMC10215460 37238132

[ece373002-bib-0114] Yip, S. J. S. , M.‐A. Rich , and C. R. Dickman . 2015. “Diet of the Feral Cat, *Felis catus*, in Central Australian Grassland Habitats During Population Cycles of Its Principal Prey.” Mammal Research 60, no. 1: 39–50. 10.1007/s13364-014-0208-7.

