# Supplementary material for: Boom‐Bust Dynamics Drive Community‐Wide Dietary Structuring in Desert‐Dwelling Raptors
Source: Ecol Evol. 2026 Jan 30;16(2):e73002. doi: 10.1002/ece3.73002 (PMC12859166; doi:10.1002/ece3.73002)
Supplement: Supplementary file 2 — Appendix S2: ece373002‐sup‐0002‐AppendixS2.docx. [file ECE3-16-e73002-s001.docx]

**Appendix**

Table S1: Mammal species considered as small and large mammal species for analyses

| Group | Common name | Species name |
| --- | --- | --- |
| Small mammal | Brush-tailed mulgara | *Dasycercus blythi* |
|  | Forrest’s mouse | *Leggadina forresti* |
|  | House mouse | *Mus musculus* |
|  | Spinifex hopping-mouse | *Notomys alexis* |
|  | Fawn hopping-mouse | *Notomys cervinus* |
|  | European rabbit | *Oryctolagus cuniculus* |
|  | Desert mouse | *Pseudomys desertor* |
|  | Sandy inland mouse | *Pseudomys hermannsburgensis* |
|  | Long-haired rat | *Rattus villosissimus* |
|  | Dunnart sp. | *Sminthopsis* spp*.* |
|  | Wongai ningaui | *Ningaui ridei* |
|  | Echidna | *Tachyglossus aculeatus* |
|  | Microbat |  |
| Large mammals | Red kangaroo | *Osphranter rufus* |
|  | Cow | *Bos taurus* |
|  | Red fox | *Vulpes vulpes* |
|  | Feral cat | *Felis catus* |
|  | Feral pig | *Sus scrofa* |

Table S2: Overall mean (+/- SE) proportions of different dietary components identified within pellets of each raptor species recorded, stratified across boom and bust periods. N = sample size (number of pellets examined).

| Period | Species group | Species | Small mammal | Large mammal | Avian | Reptile | Invertebrate | Amphibian | Other | Fish |
| --- | --- | --- | --- | --- | --- | --- | --- | --- | --- | --- |
| Boom | Accipitriform | Black Kite  (*Milvus migrans*)  N = 14 | 65.286 +/- 12.580 | 0 | 29.643 +/- 11.235 | 3.571 +/- 3.571 | 0.714 +/- 0.485 | 0 | 0.786 +/- 0.712 | 0 |
|  |  | Black-breasted Buzzard  (*Hamirostra melanosternon*)  N = 42 | 27.000 +/- 5.781 | 3.571 +/- 2.636 | 54.690 +/- 6.121 | 14.500 +/- 3.768 | 0.238 +/- 0.075 | 0 | 0 | 0 |
|  |  | Black-shouldered Kite  (*Elanus axillaris*)  N = 128 | 97.703 +/- 1.183 | 0 | 1.953 +/- 1.164 | 0.234 +/- 0.234 | 0.109 +/- 0.028 | 0 | 0 | 0 |
|  |  | Brown Goshawk  (*Accipiter fasciatus*)  N = 29 | 8.966 +/- 4.612 | 0 | 51.207 +/- 7.802 | 39.552 +/- 8.011 | 0.276 +/- 0.084 | 0 | 0 | 0 |
|  |  | Collared Sparrowhawk  (*Accipiter cirrocephalus*)  N = 15 | 0 | 0 | 99.800 +/- 0.107 | 0 | 0.200 +/- 0.107 | 0 | 0 | 0 |
|  |  | Letter-winged Kite  (*Elanus scriptus*)  N = 62 | 90.710 +/- 2.073 | 0 | 2.242 +/- 0.786 | 0 | 7.032 +/- 1.995 | 0 | 0.016 +/- 0.016 | 0 |
|  |  | Spotted Harrier  (*Circus assimilis*)  N = 21 | 59.714 +/- 8.880 | 0 | 22.952 +/- 8.205 | 16.762 +/- 7.090 | 0.333 +/- 0.242 | 0.238 +/- 0.238 | 0 | 0 |
|  |  | Square-tailed Kite  (*Lophoictinia isura*)  N = 59 | 0 | 0 | 98.780 +/- 0.861 | 0.847 +/- 0.847 | 0.288 +/- 0.164 | 0 | 0.085 +/- 0.085 | 0 |
|  |  | Swamp Harrier  (*Circus approximans*)  N = 11 | 13.636 +/- 9.749 | 6.091 +/- 6.091 | 58.909 +/- 14.750 | 18.818 +/- 10.711 | 0.273 +/- 0.195 | 0 | 0 | 2.273 +/- 2.273 |
|  |  | Wedge-tailed Eagle  (*Aquila audax*)  N = 33 | 63.636 +/- 7.450 | 23.485 +/- 6.609 | 9.848 +/- 4.478 | 3.030 +/- 1.713 | 0 | 0 | 0 | 0 |
|  |  | Whistling Kite  (*Haliastur sphenurus*)  N = 15 | 43.133 +/- 11.618 | 0 | 52.200 +/- 11.923 | 2.333 +/- 2.333 | 0.333 +/- 0.159 | 0 | 0 | 2.000 +/- 1.676 |
|  | Falconiform | Australian Hobby  (*Falco longipennis*)  N = 17 | 0.882 +/- 0.882 | 0 | 97.294 +/- 1.025 | 0 | 1.235 +/- 0.466 | 0.588 +/- 0.588 | 0 | 0 |
|  |  | Black Falcon  (*Falco subniger*)  N = 47 | 6.383 +/- 3.604 | 0 | 92.851 +/- 3.590 | 0.106 +/- 0.106 | 0.660 +/- 0.319 | 0 | 0 | 0 |
|  |  | Brown Falcon  (*Falco berigora*)  N = 86 | 37.488 +/- 4.409 | 1.163 +/- 1.105 | 10.233 +/- 2.236 | 47.047 +/- 4.305 | 1.593 +/- 0.294 | 1.686 +/- 1.242 | 0.791 +/- 0.434 | 0 |
|  |  | Grey Falcon  (*Falco hypoleucos*)  N = 41 | 0.732 +/- 0.732 | 0 | 97.634 +/- 1.108 | 0.854 +/- 0.854 | 0.488 +/- 0.213 | 0 | 0.293 +/- 0.136 | 0 |
|  |  | Nankeen Kestrel  (*Falco cenchroides*)  N = 81 | 0.494 +/- 0.494 | 0 | 0 | 9.605 +/- 1.195 | 88.728 +/- 1.306 | 0 | 1.173 +/- 0.431 | 0 |
|  |  | Peregrine Falcon  (*Falco peregrinus*)  N = 14 | 3.571 +/- 3.571 | 0 | 96.214 +/- 3.557 | 0 | 0.214 +/- 0.114 | 0 | 0 | 0 |
|  | Strigiform | Barking Owl  (*Ninox connivens*)  N =27 | 82.444 +/- 4.887 | 0 | 8.889 +/- 4.661 | 3.074 +/- 1.723 | 5.000 +/- 2.505 | 0 | 0.593 +/- 0.228 | 0 |
| Bust | Accipitriform | Black Kite  (*Milvus migrans*)  N = 41 | 9.512 +/- 4.579 | 2.439 +/- 2.439 | 37.439 +/- 7.300 | 35.561 +/- 7.172 | 5.171 +/- 2.531 | 2.439 +/- 2.439 | 5.000 +/- 3.404 | 2.439 +/- 2.439 |
|  |  | Black-breasted Buzzard  (*Hamirostra melanosternon*)  N = 71 | 21.479 +/- 3.643 | 10.521 +/- 3.333 | 35.662 +/- 3.909 | 31.352 +/- 3.706 | 0.634 +/- 0.185 | 0 | 0.352 +/- 0.352 | 0 |
|  |  | Black-shouldered Kite  (*Elanus axillaris*)  N = 53 | 91.811 +/- 3.668 | 0 | 5.660 +/- 3.205 | 2.358 +/- 1.936 | 0.170 +/- 0.052 | 0 | 0 | 0 |
|  |  | Brown Goshawk  (*Accipiter fasciatus*)  N = 46 | 3.739 +/- 2.040 | 0 | 26.239 +/- 4.931 | 69.543 +/- 5.152 | 0.478 +/- 0.123 | 0 | 0 | 0 |
|  |  | Collared Sparrowhawk  (*Accipiter cirrocephalus*)  N = 24 | 0 | 0 | 91.375 +/- 5.746 | 8.333 +/- 5.763 | 0.292 +/- 0.112 | 0 | 0 | 0 |
|  |  | Letter-winged Kite  (*Elanus scriptus*)  N = 75 | 92.907 +/- 1.969 | 0 | 2.973 +/- 1.232 | 2.347 +/- 1.223 | 1.213 +/- 0.494 | 0 | 0.560 +/- 0.256 | 0 |
|  |  | Little Eagle  (*Hieraaetus morphnoides*)  N = 44 | 34.932 +/- 6.167 | 9.091 +/- 3.963 | 18.636 +/- 4.812 | 35.182 +/- 5.870 | 1.932 +/- 0.978 | 0 | 0.227 +/- 0.227 | 0 |
|  |  | Spotted Harrier  (*Circus assimilis*)  N = 24 | 9.667 +/- 5.812 | 0 | 42.125 +/- 8.800 | 47.083 +/- 8.921 | 0.500 +/- 0.233 | 0 | 0.625 +/- 0.458 | 0 |
|  |  | Swamp Harrier  (*Circus approximans*)  N = 22 | 29.500 +/- 9.415 | 4.545 +/- 3.137 | 39.864 +/- 9.912 | 24.091 +/- 8.222 | 0.500 +/- 0.253 | 1.500 +/- 1.500 | 0 | 0 |
|  |  | Wedge-tailed Eagle  (*Aquila audax*)  N = 42 | 46.905 +/- 6.810 | 25.119 +/- 6.379 | 16.667 +/- 4.899 | 11.190 +/- 3.321 | 0.119 +/- 0.119 | 0 | 0 | 0 |
|  |  | Whistling Kite  (*Haliastur sphenurus*)  N = 19 | 3.368 +/- 2.693 | 7.895 +/- 5.752 | 71.263 +/- 9.174 | 13.789 +/- 6.517 | 3.263 +/- 1.973 | 0 | 0.421 +/- 0.421 | 0 |
|  | Falconiform | Australian Hobby  (*Falco longipennis*)  N = 34 | 2.912 +/- 2.912 | 0 | 94.471 +/- 2.961 | 0.588 +/- 0.588 | 1.882 +/- 0.547 | 0 | 0.147 +/- 0.147 | 0 |
|  |  | Black Falcon  (*Falco subniger*)  N = 21 | 9.524 +/- 6.564 | 0 | 80.286 +/- 8.712 | 9.429 +/- 6.499 | 0.762 +/- 0.257 | 0 | 0 | 0 |
|  |  | Brown Falcon  (*Falco berigora*)  N = 72 | 14.00 +/- 3.433 | 1.389 +/- 0.975 | 13.083 +/- 3.148 | 68.722 +/- 4.078 | 2.389 +/- 0.705 | 0 | 0.417 +/- 0.192 | 0 |
|  |  | Grey Falcon  (*Falco hypoleucos*)  N = 30 | 1.167 +/- 1.167 | 0 | 94.700 +/- 3.305 | 3.167 +/- 3.167 | 0.600 +/- 0.282 | 0 | 0.367 +/- 0.122 | 0 |
|  |  | Nankeen Kestrel  (*Falco cenchroides*)  N = 60 | 0 | 0 | 0 | 10.000 +/- 1.182 | 89.467 +/- 1.163 | 0 | 0.533 +/- 0.263 | 0 |
|  |  | Peregrine Falcon  (*Falco peregrinus*)  N = 5 | 0 | 0 | 99.800 +/- 0.200 | 0 | 0.200 +/- 0.200 | 0 | 0 | 0 |
|  | Strigiform | Barking Owl  (*Ninox connivens*)  N = 19 | 79.211 +/- 7.348 | 0 | 6.474 +/- 4.238 | 7.632 +/- 5.276 | 3.526 +/- 1.474 | 0 | 3.158 +/- 2.618 | 0 |

Table S3: Mvabund output of post-hoc tests undertaken assessing the influence of raptor species group, period (boom / bust) and their interaction with the raptors' dietary components.

| Variable | Deviance | p-value |
| --- | --- | --- |
| Multivariate tests | | |
| Species group | 391.600 | 0.001 |
| Period | 56.700 | 0.001 |
| Species group * Period | 31.700 | 0.001 |
| Variable | Deviance | p-value |
| Small mammal | | |
| Species group | 81.886 | 0.001 |
| Period | 9.956 | 0.023 |
| Species group * Period | 1.736 | 0.201 |
| Large mammal | | |
| Species group | 11.141 | 0.038 |
| Period | 0.936 | 0.815 |
| Species group * Period | 0.201 | 0.990 |
| Avian | | |
| Species group | 12.440 | 0.024 |
| Period | 0.736 | 0.833 |
| Species group * Period | 0.246 | 0.990 |
| Reptile | | |
| Species group | 6.644 | 0.177 |
| Period | 30.940 | 0.001 |
| Species group * Period | 6.395 | 0.129 |
| Invertebrate | | |
| Species group | 271.154 | 0.001 |
| Period | 0.002 | 0.975 |
| Species group * Period | 0.164 | 0.990 |
| Amphibian | | |
| Species group | 0.509 | 0.623 |
| Period | 0.349 | 0.917 |
| Species group * Period | 3.646 | 0.403 |
| Other | | |
| Species group | 3.957 | 0.425 |
| Period | 13.677 | 0.009 |
| Species group * Period | 19.277 | 0.006 |
| Fish | | |
| Species group | 3.880 | 0.425 |
| Period | 0.051 | 0.975 |
| Species group * Period | 0 | 1 |

Table S4: Mvabund output of post-hoc tests undertaken assessing the influence of raptor species and the interaction of species and period (boom / bust) with raptor dietary components.

| Variable | Deviance | p-value |
| --- | --- | --- |
| Multivariate tests | | |
| Species | 2423.700 | 0.001 |
| Species * Period | 343.300 | 0.001 |
| Variable | Deviance | p-value |
| Small mammal | | |
| Species | 505.861 | 0.001 |
| Species* Period | 70.583 | 0.001 |
| Large mammal | | |
| Species | 119.154 | 0.001 |
| Species* Period | 6.736 | 0.626 |
| Avian | | |
| Species | 508.218 | 0.001 |
| Species* Period | 15.552 | 0.626 |
| Reptile | | |
| Species | 303.543 | 0.001 |
| Species* Period | 146.61 | 0.001 |
| Invertebrate | | |
| Species | 827.164 | 0.001 |
| Species* Period | 59.644 | 0.001 |
| Amphibian | | |
| Species | 18.113 | 0.113 |
| Species* Period | 7.042 | 0.626 |
| Other | | |
| Species | 121.686 | 0.001 |
| Species* Period | 32.351 | 0.026 |
| Fish | | |
| Species | 20.059 | 0.073 |
| Species* Period | 4.831 | 0.626 |

Table S5: Mvabund output of post-hoc tests undertaken assessing the influence of raptor species, period (boom / bust) and the interaction of species and period on small mammals as raptor dietary components.

| Variable | Deviance | p-value |
| --- | --- | --- |
| Multivariate tests | | |
| Period | 8.5 | 0.013 |
| Species | 532.100 | 0.001 |
| Species * Period | 101.300 | 0.011 |
| Variable | Deviance | p-value |
| *Rattus villosissimus* | | |
| Period | 0.475 | 0.577 |
| Species | 85.241 | 0.001 |
| Species * Period | 45.406 | 0.011 |
| *Pseudomys hermannsburgensis* | | |
| Period | 0.059 | 0.901 |
| Species | 44.643 | 0.001 |
| Species * Period | 12.220 | 0.324 |
| *Leggadina forresti* | | |
| Period | 0.014 | 0.901 |
| Species | 50.071 | 0.001 |
| Species * Period | 10.201 | 0.384 |
| *Sminthopsis* spp. | | |
| Period | 3.097 | 0.059 |
| Species | 20.937 | 0.022 |
| Species * Period | 11.670 | 0.324 |
| *Notomys cervinus* | | |
| Period | 0.071 | 0.901 |
| Species | 34.222 | 0.001 |
| Species * Period | 8.383 | 0.580 |
| *Mus musculus* | | |
| Period | 1.263 | 0.283 |
| Species | 113.096 | 0.001 |
| Species * Period | 3.740 | 0.997 |
| *Notomys alexis* | | |
| Period | 0.323 | 0.657 |
| Species | 20.294 | 0.022 |
| Species * Period | 3.968 | 0.997 |
| *Dasycercus blythi* | | |
| Period | 0.445 | 0.577 |
| Species | 20.294 | 0.022 |
| Species * Period | 2.400 | 0.997 |
| *Oryctolagus cuniculus* | | |
| Period | 0.837 | 0.283 |
| Species | 111.236 | 0.001 |
| Species * Period | 3.197 | 0.997 |
| *Pseudomys desertor* | | |
| Period | 0.153 | 0.867 |
| Species | 27.284 | 0.001 |
| Species * Period | 0.100 | 0.997 |
| *Tachyglossus aculeatus* | | |
| Period | 1.751 | 0.283 |
| Species | 4.671 | 0.278 |
| Species * Period | 0.008 | 0.997 |

Table S6: Mvabund output of post-hoc tests undertaken assessing the influence of species movement status and the interaction of species status and period on dietary components.

| Variable | Deviance | p-value |
| --- | --- | --- |
| Multivariate tests | | |
| Species status | 481.000 | 0.001 |
| Species status * Period | 176.600 | 0.001 |
| Variable | Deviance | p-value |
| Small mammal | | |
| Species status | 4.371 | 0.234 |
| Species status * Period | 18.002 | 0.001 |
| Large mammal | | |
| Species status | 2.201 | 0.505 |
| Species status * Period | 5.361 | 0.273 |
| Avian | | |
| Species status | 87.496 | 0.001 |
| Species status * Period | 13.401 | 0.004 |
| Reptile | | |
| Species status | 21.458 | 0.006 |
| Species status * Period | 60.678 | 0.001 |
| Invertebrate | | |
| Species status | 354.383 | 0.001 |
| Species status * Period | 44.968 | 0.001 |
| Amphibian | | |
| Species status | 0.234 | 0.793 |
| Species status * Period | 4.280 | 0.404 |
| Other | | |
| Species status | 7.966 | 0.172 |
| Species status * Period | 28.464 | 0.001 |
| Fish | | |
| Species status | 2.889 | 0.505 |
| Species status * Period | 1.454 | 0.621 |

Table S7: GAM output assessing the interaction of species group and year on average dietary component composition.

| Variable | e.d.f | Reference e.d.f | χ^2^ | p-value |
| --- | --- | --- | --- | --- |
| Accipitriform group – deviance explained 14.8% | | | | |
| s(Year): Amphibian | 1.000 | 1.000 | 0.001 | 0.971 |
| s(Year): Avian | 1.686 | 1.901 | 3.443 | 0.227 |
| s(Year): Fish | 1.000 | 1.000 | 0.001 | 0.981 |
| s(Year): Invertebrate | 1.000 | 1.000 | 0.005 | 0.942 |
| s(Year): Large mammal | 1.000 | 1.000 | 0.614 | 0.433 |
| s(Year): Other | 1.000 | 1.000 | 0.383 | 0.536 |
| s(Year): Reptile | 1.748 | 1.937 | 4.331 | 0.158 |
| s(Year): Small mammal | 1.881 | 1.986 | 15.337 | **0.0003** |
| Falconiform group – deviance explained 16.6% | | | | |
| s(Year): Amphibian | 1.000 | 1.000 | 0.070 | 0.791 |
| s(Year): Avian | 1.663 | 1.887 | 14.335 | **0.001** |
| s(Year): Fish | 1.000 | 1.000 | 0 | 1.000 |
| s(Year): Invertebrate | 1.000 | 1.000 | 0.574 | 0.449 |
| s(Year): Large mammal | 1.000 | 1.000 | 0.235 | 0.628 |
| s(Year): Other | 1.000 | 1.000 | 0.015 | 0.903 |
| s(Year): Reptile | 1.000 | 1.000 | 1.626 | 0.202 |
| s(Year): Small mammal | 1.000 | 1.000 | 0.550 | 0.458 |

Table S8: GAM output assessing the interaction of species movement status and year on average dietary component composition.

| Variable | e.d.f | Reference e.d.f | χ^2^ | p-value |
| --- | --- | --- | --- | --- |
| Locally nomadic– deviance explained 13.9% | | | | |
| s(Year): Amphibian | 1.000 | 1.000 | 0.029 | 0.865 |
| s(Year): Avian | 1.726 | 1.925 | 13.776 | **0.003** |
| s(Year): Fish | 1.000 | 1.000 | 0.002 | 0.962 |
| s(Year): Invertebrate | 1.000 | 1.000 | 0.132 | 0.717 |
| s(Year): Large mammal | 1.000 | 1.000 | 0.047 | 0.828 |
| s(Year): Other | 1.000 | 1.000 | 0.570 | 0.450 |
| s(Year): Reptile | 1.000 | 1.000 | 0.132 | 0.717 |
| s(Year): Small mammal | 1.795 | 1.958 | 13.090 | **0.001** |
| Nomadic – deviance explained 6.52% | | | | |
| s(Year): Amphibian | 1.000 | 1.000 | 0.006 | 0.937 |
| s(Year): Avian | 1.000 | 1.000 | 0.607 | 0.436 |
| s(Year): Fish | 1.000 | 1.000 | 0.004 | 0.948 |
| s(Year): Invertebrate | 1.000 | 1.000 | 0.432 | 0.511 |
| s(Year): Large mammal | 1.001 | 1.001 | 0.003 | 0.962 |
| s(Year): Other | 1.000 | 1.000 | 0.034 | 0.854 |
| s(Year): Reptile | 1.000 | 1.000 | 2.075 | 0.150 |
| s(Year): Small mammal | 1.000 | 1.000 | 0.032 | 0.857 |
| Resident – deviance explained 8% | | | | |
| s(Year): Amphibian | 1.000 | 1.000 | 0.165 | 0.684 |
| s(Year): Avian | 1.000 | 1.000 | 0.576 | 0.448 |
| s(Year): Fish | 1.000 | 1.000 | 0 | 1.000 |
| s(Year): Invertebrate | 1.000 | 1.000 | 0.524 | 0.469 |
| s(Year): Large mammal | 1.000 | 1.000 | 0.023 | 0.879 |
| s(Year): Other | 1.000 | 1.000 | 0.003 | 0.959 |
| s(Year): Reptile | 1.000 | 1.000 | 1.091 | 0.296 |
| s(Year): Small mammal | 1.000 | 1.000 | 4.323 | **0.038** |


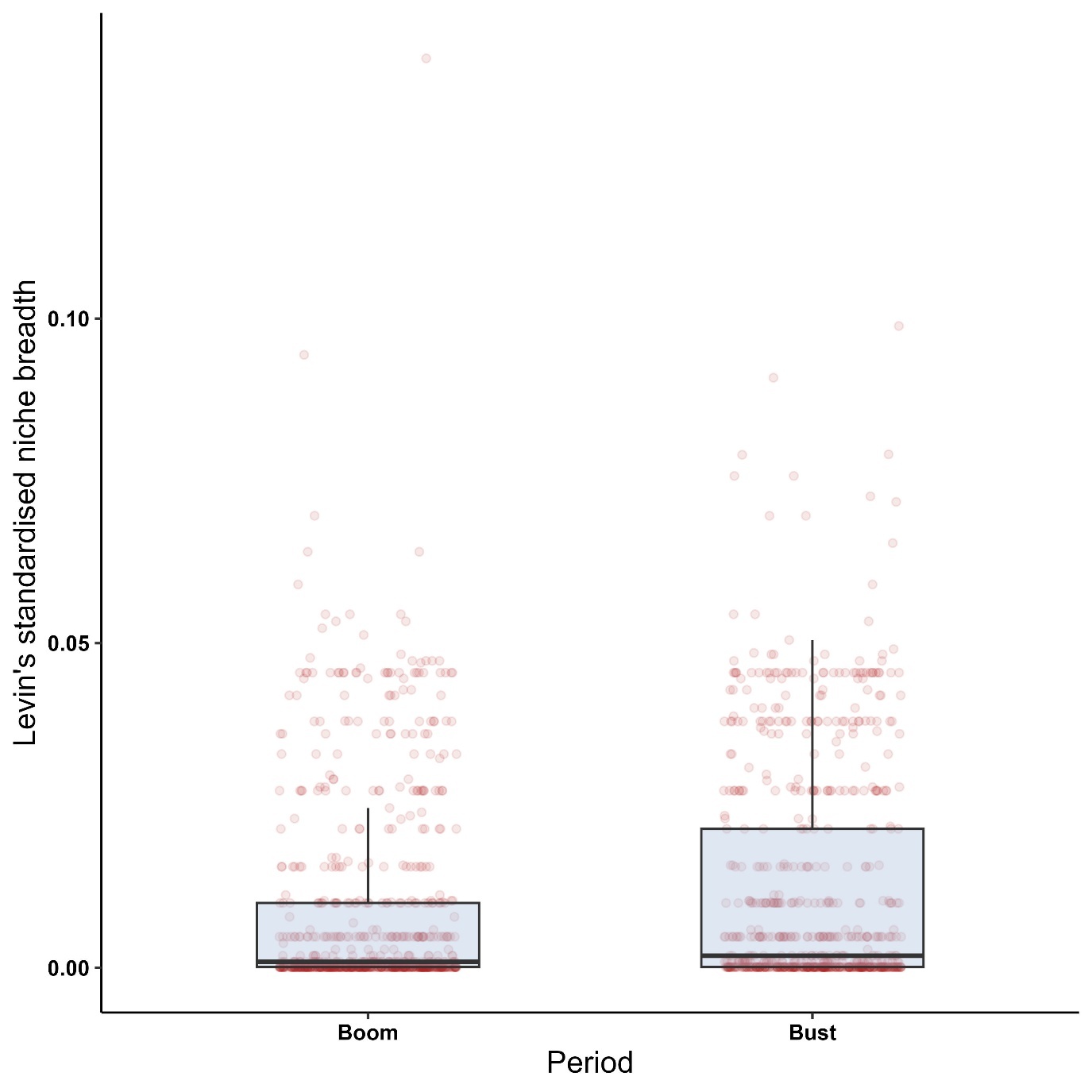
Figure S1: Levins’ standardised dietary niche breadth, calculated across pellets from all raptor species, shown as box-and-whisker plots between boom-and-bust periods.


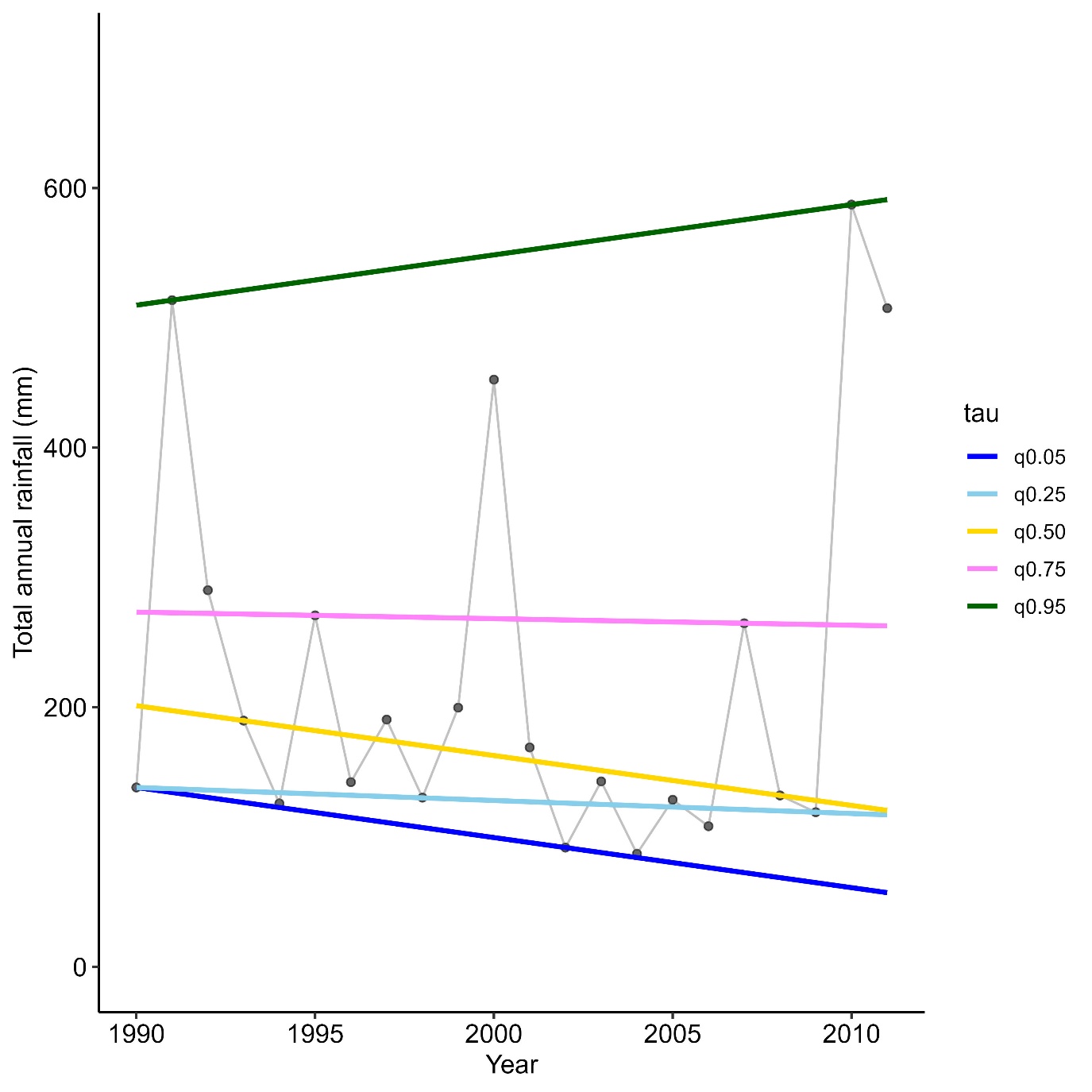


Figure S2: Quantile regression plot of total annual rainfall (mm) from the study area across the entire monitoring period.
